# Loss of voltage-gated hydrogen channel 1 expression reveals heterogeneous metabolic adaptation to intracellular acidification by T cells

**DOI:** 10.1172/jci.insight.147814

**Published:** 2022-05-23

**Authors:** David Coe, Thanushiyan Poobalasingam, Hongmei Fu, Fabrizia Bonacina, Guosu Wang, Valle Morales, Annalisa Moregola, Nico Mitro, Kenneth C.P. Cheung, Eleanor J. Ward, Suchita Nadkarni, Dunja Aksentijevic, Katiuscia Bianchi, Giuseppe Danilo Norata, Melania Capasso, Federica M. Marelli-Berg

**Affiliations:** 1 William Harvey Research Institute, Barts and The London School of Medicine and Dentistry, Queen Mary University of London, Charterhouse Square, London, United Kingdom. Research Institute, Queen Mary University of London, London, United Kingdom.; 2 Centre for Inflammation and Therapeutic Innovation, Queen Mary University of London, Charterhouse Square, London, United Kingdom.; 3 Department of Pharmacological and Biomolecular Sciences, University of Milan, Milan, Italy.; 4 Centre for Molecular Oncology, Barts Cancer Institute, Queen Mary University of London, Charterhouse Square, London, United Kingdom.; 5 Center for Neurodegenerative Diseases (DZNE) within the Helmholtz Association, Bonn, Germany.

**Keywords:** Immunology, Adaptive immunity

## Abstract

Voltage-gated hydrogen channel 1 (Hvcn1) is a voltage-gated proton channel, which reduces cytosol acidification and facilitates the production of ROS. The increased expression of this channel in some cancers has led to proposing Hvcn1 antagonists as potential therapeutics. While its role in most leukocytes has been studied in depth, the function of Hvcn1 in T cells remains poorly defined. We show that Hvcn1 plays a nonredundant role in protecting naive T cells from intracellular acidification during priming. Despite sharing overall functional impairment in vivo and in vitro, Hvcn1-deficient CD4^+^ and CD8^+^ T cells display profound differences during the transition from naive to primed T cells, including in the preservation of T cell receptor (TCR) signaling, cellular division, and death. These selective features result, at least in part, from a substantially different metabolic response to intracellular acidification associated with priming. While Hvcn1-deficient naive CD4^+^ T cells reprogram to rescue the glycolytic pathway, naive CD8^+^ T cells, which express high levels of this channel in the mitochondria, respond by metabolically compensating mitochondrial dysfunction, at least in part via AMPK activation. These observations imply heterogeneity between adaptation of naive CD4^+^ and CD8^+^ T cells to intracellular acidification during activation.

## Graphical abstract

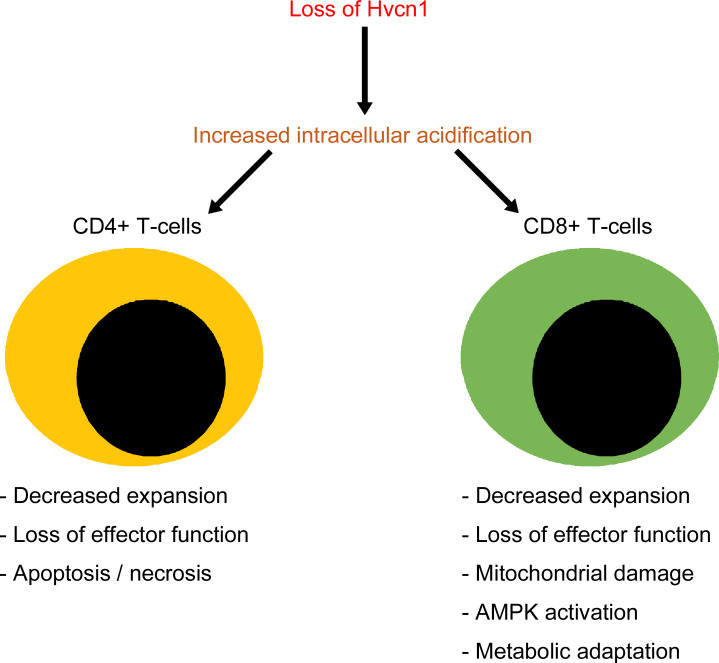

## Introduction

Acidification of the extracellular environment and the cytoplasm is associated with many physiological and pathological conditions, such as hypoxia and tumorigenesis (1–3). Intracellular acidification also plays an important role in modulating the function of immune cells, including lymphocytes. Acidic conditions (low pH) have been shown to reduce proliferation and function of human and mouse T cells as a consequence of blunted glycolysis (4–7), while restoration of pH to physiological levels rescues T cell function (4, 8).

Cells have many ways to relieve intracellular acidification through exchangers and cotransporters (9). Among these, proton channels are particularly useful when nutrient availability is limited, as they neither require ATP nor depend on, or affect, the concentration of other electrolytes.

Voltage-gated hydrogen channel 1 (Hvcn1) is a voltage-gated proton channel that opens in response to changes in membrane potential to selectively remove protons from the cytoplasm (10, 11). Hvcn1 has 2 key functions: first, it responds to low intracellular pH (pH^i^) by reducing cytosol acidification (12–14); and second, Hvcn1 facilitates the production of ROS by NADPH oxidases (15).

HVCN1 is expressed by a number of cells, including human spermatozoa and airway epithelial cells (16). Importantly, increased expression of this channel has been reported in cancer, where it promotes survival in the acidic environment (16). This has recently given scope to the development of Hvcn1 antagonists, also supported by the reports that global Hvcn1 do not display any severe spontaneous pathology and have a normal life span (16).

The function of this channel has been studied in relative depth in leukocytes. In B cells and phagocytes, Hvcn1 is associated with NADPH oxidases on the cell surface, and in endosomes/phagosomes where it facilitates the generation of ROS by re-equilibrating the membrane potential. ROS are required in B cells for signaling via the B cell receptor (BCR) (17) and in phagocytes for phagosome bacterial destruction (12, 18–20).

The role of Hvcn1 in T cells remains poorly defined and somehow contentious. Hvcn1 protein has been shown to be expressed by naive T cells (21) and the presence of Hvcn1 has been assumed in activated T cells, which exhibit the voltage-activated proton currents that are characteristic of Hvcn1 (22). Germline *Hvcn1* KO mice had increased frequency of CD44^hi^ CD4^+^ and CD8^+^ T cells and presented nephritis and splenomegaly with aging (21). However, inhibition of HVCN1 in leukemic Jurkat T cells decreased pH^i^ and caused cell death by apoptosis (21, 23).

Here, we show that Hvcn1 plays a nonredundant role in regulating intracellular acidification during T cell activation, affecting T cell receptor (TCR) signaling, proliferation, and effector function. Loss of Hvcn1 expression differentially affects activation-induced metabolic reprogramming of CD4^+^ and CD8^+^ T cells, leading to distinct functional consequences.

## Results

### Lack of the proton channel Hvcn1 differentially affects CD4^+^ and CD8^+^ T cell responses.

**Figure 1:**
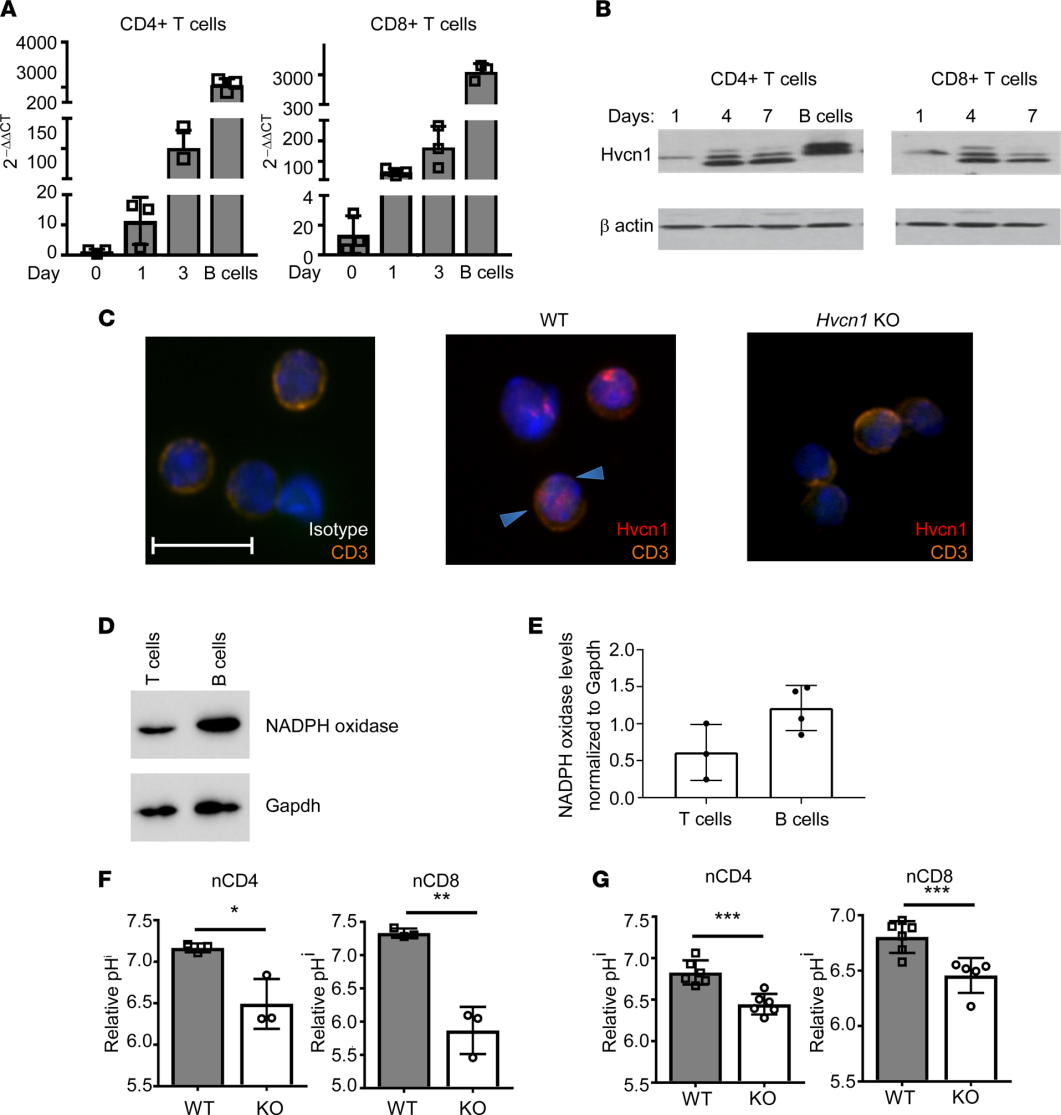
The proton channel Hvcn1 is expressed by T lymphocytes and regulates intracellular acidity. T cells were purified from spleen and lymph nodes (LN) of WT mice and stimulated with plate-bound anti-CD3 (1 μg/mL) and anti-CD28 (5 μg/mL) with 20 U/mL IL-2 for the indicated number of days. Expression of *Hvcn1* gene and protein was measured by quantitative PCR (qPCR) and Western blot in CD4^+^ and CD8^+^ T cell subsets (**A** and **B**, respectively). (**C**) LN T cells from WT and Hvcn1-deficient mice were stained with DAPI (blue), anti-CD3 (orange) and anti-Hvcn1 (red) Abs and visualized by confocal microscopy. Scale bar: 10 μm. (**D** and **E**) Total T cells and B cells were isolated from WT mice (*n* = 3) and the protein extract resolved by SDS gel electrophoresis. NADPH oxidase levels were normalized to GAPDH levels. (**F** and **G**) Relative pH^i^ of naive (n)CD4^+^ and nCD8^+^ (*n* = 3, **F**) and Ab-activated (day 4, **G**) CD4^+^ and CD8^+^ (*n* = 6) WT and Hvcn1-deficient (KO) T cells was calculated by staining with pHRodo. Results are presented as mean ± SD. *P < 0.05, **P < 0.01, ***P < 0.005; 2-tailed Student’s *t* test.

To study the role of Hvcn1 in T lymphocytes, we first analyzed the expression of this molecule in naive and anti–CD3/anti–CD28-activated T cells. Naive T cells expressed low levels of Hvcn1, which was upregulated following activation both at the transcriptional and protein levels (Figure 1, A and B), unlike in B cells, which express high levels of Hvcn1 when resting and downregulate it following activation (17). Confocal microscopy experiments showed that T cells express Hvcn1 on the surface and possibly intracellularly (Figure 1C). In addition, T cells expressed lower levels of NADPH oxidase compared with B cells (Figure 1, D and E), in which this enzyme is associated with Hvcn1 at the cell surface (17).

As a key function of Hvcn1 is to prevent intracellular acidification, we first compared the pH^i^ of WT and Hvcn1-deficient T cells after labeling with the pH-sensitive dye pHRodo. As shown in Figure 1, F and G, both Hvcn1-deficient naive (n) and CD3/CD28-activated (a) CD4^+^ and CD8^+^ T cells displayed significantly lower pH^i^ than their WT counterpart. Intracellular acidification was reduced in activated T cells compared with naive Hvcn1-deficient T cells.

A baseline analysis of Hvcn1-deficient mice did not reveal any significant alteration of T lymphocyte thymic maturation (Supplemental Figure 1A and Supplemental Figure 2; supplemental material available online with this article; https://doi.org/10.1172/jci.insight.147814DS1 for flow cytometry analysis gating).

The number of total Hvcn1-null T cells detected in the secondary lymphoid organs of Hvcn1-deficient T cells tended to decrease (Supplemental Figure 1B). In addition, when total memory T cell populations were analyzed, a significant decrease in the number of CD44 high memory T cells was observed, especially in Hvcn1-deficient CD4^+^ T cells and, less prominently, in the CD8^+^ memory T cell subset (Supplemental Figure 1, C and D). Although further analysis of T cell subsets did not reveal significant differences, there was an overall decreasing trend in HVNC1-null T cells (Supplemental Figure 1, E and F). Tregs were equally represented and functional (Supplemental Figure 1, G and H).

**Figure 2:**
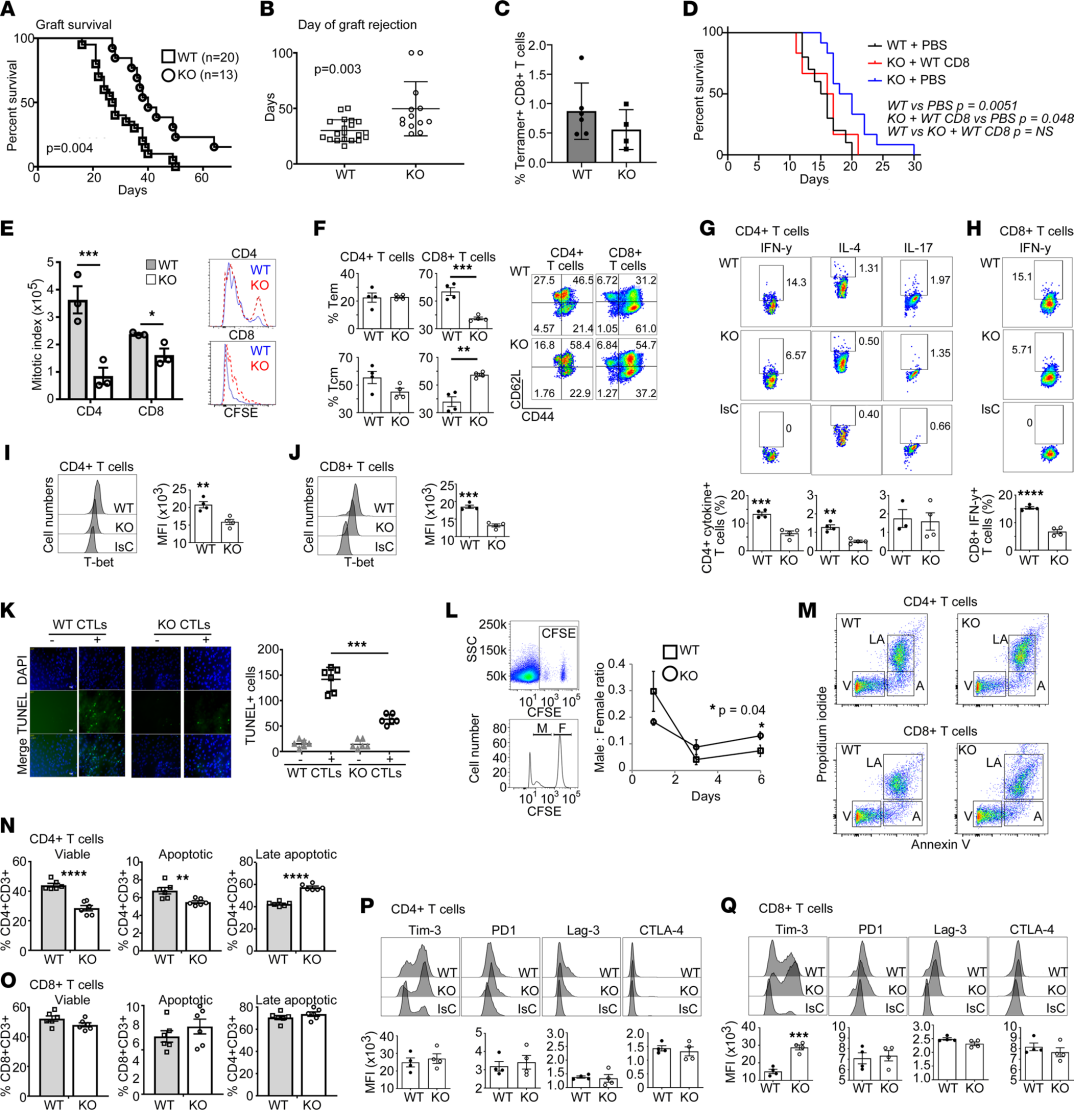
Functional features of Hvcn1-deficient T cells. (**A** and **B**) WT male-derived skin rejection by WT (*n* = 20) and Hvcn1-deficient (KO, *n* = 13) female mice ± SD. (**C**) Percentage of tetramer-positive CD8^+^ T cells in female mice after male-skin rejection. (**D**) Survival of B6Kd skin in WT (*n* = 8), Hvcn1-deficient (KO, *n* = 10) mice, and Hvcn1-deficient mice CD8^+^ T cell-depleted/repleted with 5 × 10^5^ WT cells (*n* = 10). (**E**) Cell Trace Violet-labeled naive CD4^+^ and CD8^+^ T cell proliferation. (**F**) T cell subsets after proliferation. Representative histograms and bar charts of mean data (*n* = 3, *n* = 3) are displayed ± SD. (**G** and **H**) Cytokine production and (**I** and **J**) T-bet expression measured in T cells ± SD (*n* = 4) with representative dot plots. (**K**) TUNEL assay of Balb/C-derived IFN-γ–activated ECs cocultured for 5 hours with WT or Hvcn1-deficient Ab-activated CD8^+^ T cells. Representative images and mean number of apoptotic endothelial cells (ECs) are shown ± SD (*n* = 5). Scale bar: 20 μm. (**L**) In vivo killing of female (F) or male (M) WT splenocytes stained with high and low CFSE concentrations by WT or Hvcn1-deficient females. Representative plot and histogram of differentially labeled splenocytes and proportion of CFSE hi (♀) to CFSE lo (♂) cells calculated 1 day later ± SD. (**M**–**O**) Viability of WT or Hvcn1-deficient T cells. Representative dot plots (M) and bar charts of mean apoptotic (A), late-apoptotic (LA), and viable (V) T cell proportions (**N** and **O**) ± SD (*n* = 6). (**P** and **Q**) Cell marker expression by Ab-stimulated CD4^+^ or CD8^+^ T cells. Results presented as bar charts ± SD (*n* = 4), with representative histograms. **A** and **D,** log-rank (Mantel-Cox) test. **B**, **C**, and **E**–**Q**, 2-tailed Student’s *t* test; *P < 0.05, **P < 0.01, ***P < 0.005, ****P < 0.001.

To test whether these differences were relevant to a physiological T cell response, WT and Hvcn1-deficient female mice were grafted with WT male skin. This model was chosen because transplant rejection is associated with acidification of the grafted tissue (24). In addition, the rejection of skin expressing the male HY antigens is strictly T cell-dependent and requires efficient activation of HY-specific CD4^+^ and CD8^+^ naive T cells (25, 26). As shown in Figure 2, A and B, the rejection of HY-mismatched skin grafts by Hvcn1-deficient recipients was significantly delayed compared with that by WT recipients. Tetramer analysis of CD8^+^ T cells from graft-draining lymph nodes showed a downward trend in the number of HY-tetramer^+^ Hvcn1-deficient CD8^+^ T cells, albeit not significant (Figure 2C). The causative role of T cell Hvcn1 deficiency in delayed graft rejection was further confirmed by experiments in which B6Kd-expressing skin was grafted on Hvcn1-deficient mice previously depleted of CD8^+^ T cells and reconstituted with WT CD8^+^ T cells, which rejected skin grafts with a similar kinetic as WT recipients (Figure 2D).

Although we have chosen to use germline Hvcn1-deficient mice to better reflect clinical settings where an antagonist is administered systemically, we also aimed to rule out defective antigen presentation due to a lack of Hvcn1 expression. As shown in Supplemental Figure 3A, the rejection of the Hvcn1-deficient male skin graft by WT female mice was similar to that of WT skin grafts. Previous studies show that loss of Hvcn1 in DCs can be compensated by vacuolar-type ATPase (V-ATPase) (27). Further, HY-presenting WT and Hvcn1-deficient DCs elicited a comparable proliferative response by HY-specific, TCR-transgenic T cells in vitro (Supplemental Figure 3B) and displayed a similar phenotype upon maturation (Supplemental Figure 3C).

Therefore, we focused on the functional properties of Hvcn1-deficient T cells using in vitro and ex vivo approaches.

In vitro, the proliferation of Hvcn1-deficient Ab-activated T cells was significantly reduced compared with WT counterparts (Figure 2E), with CD4^+^ T cells showing a more pronounced defect, despite displaying similar phenotypes (Supplemental Figure 3, D and E). In addition, upon in vitro activation, Hvcn1-deficient CD8^+^, but not CD4^+^, T cells preferentially displayed a central memory phenotype compared with their WT counterparts (Figure 2F). Differentiation into IFN-γ–producing Th1 was also impaired in Hvcn1-deficient CD4^+^ and CD8^+^ T cells (Figure 2, G and H), with only marginal reduction of IL-4 production by Hvcn1-deficient CD4^+^ T cells activated in Th2-inducing conditions. Accordingly, both Hvcn1-deficient CD4^+^ and CD8^+^ T cells displayed a significant reduction in the expression of the transcription factor T-bet (Figure 2, I and J).

Hvcn1-deficient CD8^+^ T cells showed impaired killing of target cells in vitro and in vivo, as evaluated by TUNEL assay and by monitoring the relative decrease of circulating adoptively transferred male (antigenic) compared with female (nonantigenic) splenocytes (labeled with different concentrations of CFSE) in immunized Hvcn1-deficient and WT female recipients (Figure 2, K and L, respectively). In this assay, the decrease of labeled male, compared with female, splenocytes, indicates specific male cell killing by recipient T cells.

Importantly, Hvcn1-deficient CD4^+^ and CD8^+^ T cells displayed a substantially different susceptibility to activation-induced cell death. Following Ab activation, a significantly larger fraction of activated Hvcn1-deficient CD4^+^ T cells underwent apoptosis (Figure 2, M and N). In contrast, we observed no differences between Hvcn1-deficient and WT CD8^+^ T cell viability (Figure 2, M and O). In this context, Hvcn1-deficient CD8^+^ but not CD4^+^ T cells displayed a significant upregulation of the inhibitory receptor (28) (Figure 2, P and Q) but no other markers of T cell exhaustion.

The migratory function of either naive or activated Hvcn1-deficient T cells was not affected by Hvcn1 deficiency either in vitro or in vivo (Supplemental Figure 3, F–K).

Overall, these data indicate that loss of Hvcn1 expression mainly affects the transition of naive to activated T cells and results in different outcomes in CD4^+^ and CD8^+^ T lymphocytes.

### Hvcn1 controls mTOR activation and reactive oxygen species production during T cell activation.

**Figure 3:**
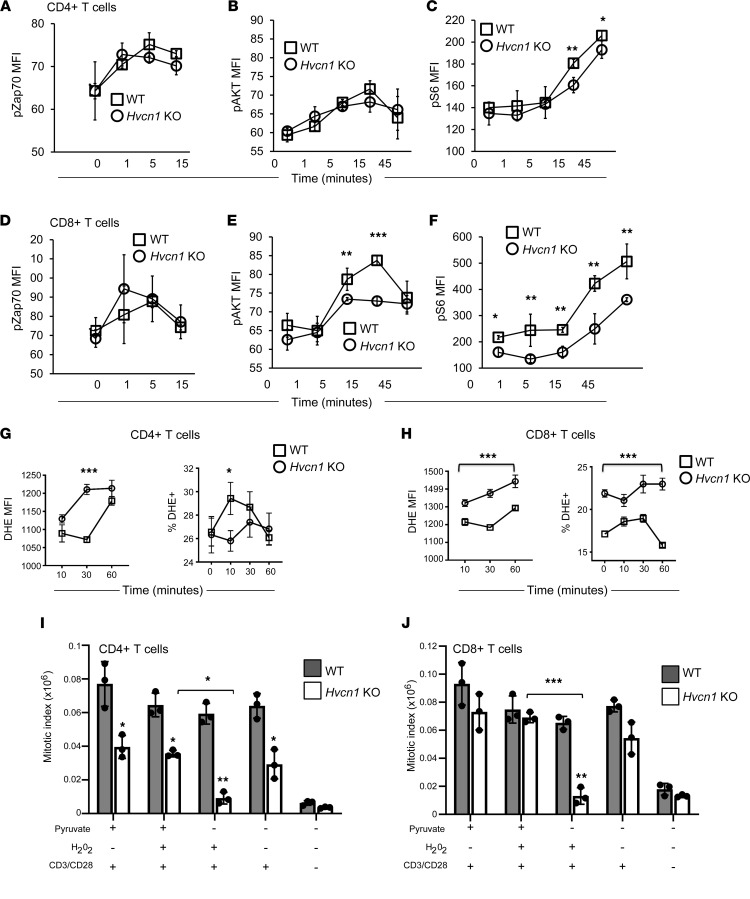
Altered TCR signaling and ROS production by Hvcn1-deficient T cells. Phosphorylation of Zap70, AKT, and S6 was measured by flow cytometry in purified naive WT or Hvcn1-deficient CD4^+^ (**A**–**C**) and CD8^+^ (**D**–**F**) T cells after Ab activation for the indicated time. Results are presented as the mean MFI ± SD. (*n* = 3 independent experiments.) (**G** and **H**) Production of superoxide was evaluated by staining nCD4^+^ and nCD8 with DHE before activating with anti-CD3/28 + 20 U/mL IL-2 at the indicated time points. Cells were analyzed by flow cytometry. Right-hand side panels show the mean MFI (level) of DHE production, while the left-hand side graph shows the mean percentage of T cells producing DHE (± SD, *n* = 4). (**I** and **J**) Purified naive WT or Hvcn1-deficient CD4^+^ or CD8^+^ T cells were stained with Cell Trace Violet and then activated in culture with or without the indicated supplements and with 20 U/mL IL-2. On day 4, cells were harvested and counted. The mitotic index was calculated as a function of the number of cells and the percentage of cells in each division, as assessed by flow cytometry. Data are presented as mean ± SD. Student’s 2-sided *t* test and 1-way ANOVA with Tukey post hoc test. *P < 0.05; **P < 0.01; ***P < 0.005.

To gain insight into the differential impact of Hvcn1 deficiency in CD4^+^ and CD8^+^ T cell activation, we investigated the intracellular events downstream of TCR engagement. In B cells, Hvcn1 is required to sustain distal BCR signaling (17). We therefore compared TCR/CD28–induced signaling in WT and Hvcn1-deficient T cells. As in B cells, proximal TCR/CD28 signaling was not affected in either subset, as indicated by equal Zap70 phosphorylation (Figure 3, A and D). Distal TCR/CD28 signaling was only partially reduced in CD4^+^ T cells, which showed a small decrease in mTORC1 signaling as indicated by diminished S6 phosphorylation (Figure 3, B and C). In contrast, Hvcn1-deficient CD8^+^ T cells displayed a severe impairment of distal TCR/CD28 signaling, with substantially reduced AKT and S6 phosphorylation compared with their WT counterpart as well as CD4-null CD4^+^ T cells (Figure 3, E and F).

In B cells, Hvcn1 sustains BCR signaling by enhancing ROS production via membrane NADPH oxidase (17). We therefore tested the production of super oxide by naive T cells labeled with dihydroethidium (DHE) and undergoing Ab stimulation. On a per-cell basis, DHE oxidation by Hvcn1-deficient CD4^+^ T cells was comparable or higher (30 minutes after stimulation) to that by WT T cells; however, the percentage of stimulated Hvcn1-deficient CD4^+^ T cells capable of oxidizing DHE was significantly lower than that of WT T cells (Figure 3G).

Surprisingly, DHE oxidation by Hvcn1-deficient CD8^+^ T cells was significantly and constitutively higher both on a per-cell basis and as a proportion of total T cells compared with WT CD8^+^ T cells (Figure 3H).

As the percentage of Hvcn1-deficient CD4^+^ T cells capable of producing ROS was significantly reduced compared with their WT counterparts, we sought to establish whether the proliferative defect of Hvcn1-deficient T cells could be rescued by peroxide addition, as has been done in T cells from mice with reduced mitochondrial ROS production (29) (Figure 3, I and J). As shown in Figure 3I, however, the mitotic index of CD4^+^ T cells was not improved by addition of peroxide in the cultures.

Despite defective TCR signaling, proliferation of Hvcn1-deficient CD8^+^ T cells was only mildly affected (Figure 3J), possibly as a compensatory effect of increased ROS production on a per-cell basis and overall (see Figure 3H). However, when T cells were cultured with peroxide in the absence of pyruvate, defective proliferation also became apparent in the Hvcn1-deficient CD8^+^ T cell subset and more profound in Hvcn1-deficient CD4^+^ T cells, while it did not affect WT T cells of either subset, indicating that in the presence of excess ROS, pyruvate availability is critical for the proliferation of T cells lacking Hvcn1 expression, possibly by feeding into the TCA cycle and mitochondrial respiration to sustain ATP production. Alternatively, pyruvate may have a direct antioxidant effect by removing H_2_O_2_ (30).

### Hvcn1-deficient naive CD8^+^ T cells display impaired mitochondrial respiration.

**Figure 4:**
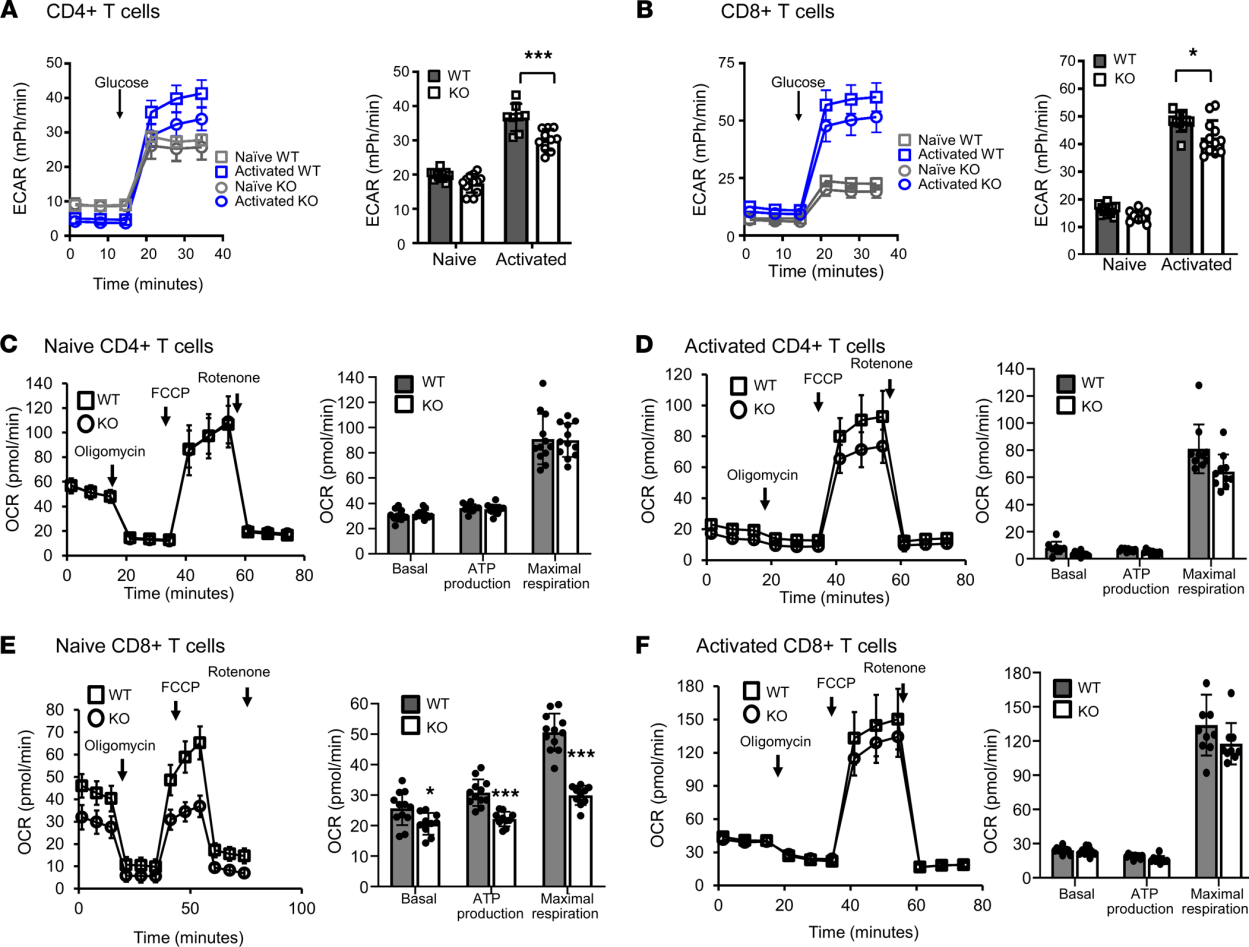
Altered metabolic responses by Hvcn1-deficient naive T cells. The ECAR (mPh/min) was measured in naive (gray) and 4-day activated (blue) Hvcn1-deficient (circles) and WT (squares) CD4^+^ (**A**) and CD8^+^ (**B**) T cells. The bar graph shows the mean peak ECAR measured in WT (gray bars) and Hvcn1-deficient (open bars) T cells (± SD; *n* = 10–12). (**C**–**F**) The OCR (pmol/min) was analyzed to evaluate OXPHOS: Naive, **C** and **E**, and activated, **D** and **F**, WT (squares) and Hvcn1-deficient (circles) CD4^+^, **C** and **D**, and CD8^+^, **E** and **F**, T cells were sequentially incubated in glucose containing media, with Oligomycin, FCCP and Antimycin plus Rotenone while the OCR was measured. The OCR was used to calculate basal and maximal respiration as well as ATP production of WT (dark gray bars) and Hvcn1-deficient (white bars) T cells (± SD; *n* = 10–12). Results are presented as mean ± SD (*n* = 5); 1-way ANOVA with Tukey post hoc test; *P < 0.05, *** *P* < 0.005.

Intracellular acidification has been shown to modify intracellular metabolism (31). Given the defective mitochondrial function, we sought to investigate the ability of Hvcn1-deficient T cells to engage energy-producing pathways following activation. As shown in Figure 4, A and B, both WT and Hvcn1-deficient naive CD4^+^ and CD8^+^ T cells were able to upregulate glycolysis following activation, albeit with a modest reduction in both subsets. Expression of the glucose transporter Glut1 was also upregulated by both CD4^+^ and CD8^+^ WT and Hvcn1-deficient T cells following Ab activation (Supplemental Figure 4, A and B), and this was accompanied by an increase of 6-NBDG [6-(*N*-(7-Nitrobenz-2-oxa-1,3-diazol-4-yl)amino)-6-Deoxyglucose] uptake (Supplemental Figure 4, C and D).

Oxidative phosphorylation (OXPHOS) was minimally affected in Hvcn1-deficient CD4^+^ T cells (Figure 4, C and D), while it was significantly impaired in naive Hvcn1-deficient CD8^+^ T cells (Figure 4E), indicative of mitochondrial impairment in these T cells. However, mitochondrial respiration appeared to be compensated in CD8^+^ T cells following Ab activation (Figure 4F).

Of note, exposing WT T cells to 2-Guanidinobenzimidazole (2GBI), a putative selective inhibitor of Hvcn1 (32) that binds the channel from the intracellular side of the membrane and acts as a potential channel blocker by accessing the core of the voltage-sensing domain when the channel is in the open conformation, not only recapitulated but also exacerbated most of the effects observed in Hvcn1-deficient T cells, independently of the subset (Supplemental Figure 5).

**Figure 5:**
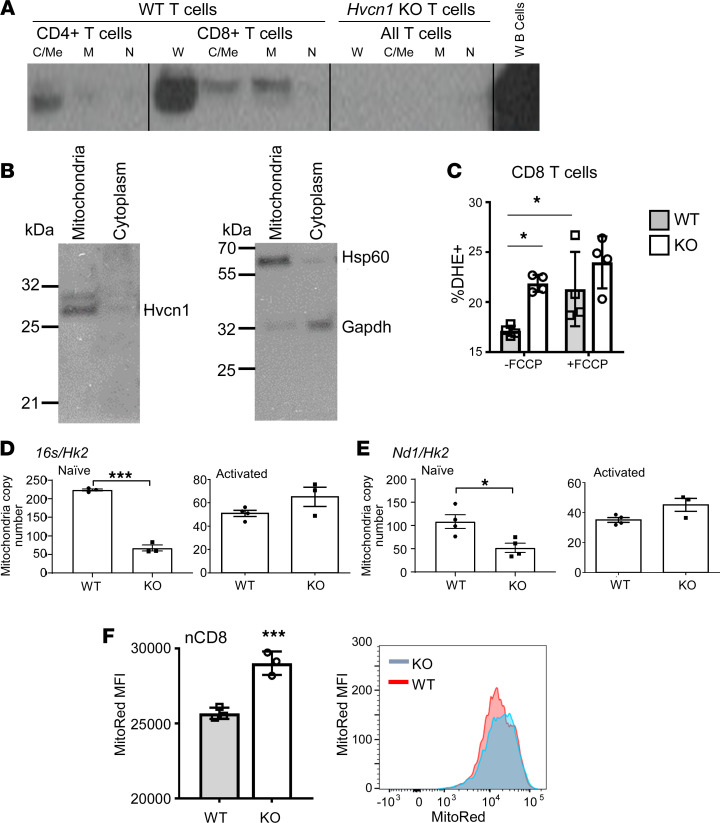
Hvcn1 is expressed by mitochondria and regulates mitochondrial electron transport chain. (**A** and **B**) Purified naive CD8^+^ WT and Hvcn1-deficient T cells were Ab-activated for 4 days, then lysed and fractionated into Cytoplasmic/Membrane (C/Me), mitochondrial (M), and nuclear (N) and were then analyzed by Western blot for the presence of Hvcn1, Hsp60, and Gapdh proteins. (**C**) Naive WT and Hvcn1-deficient CD8^+^ T cells were incubated with or without FCCP for 5 minutes before being stained with DHE and analyzed by flow cytometry. The mean percentage of DHE^+^ T cells is shown (± SD, *n* = 4). (**D** and **E**) Total DNA was isolated from naive and Ab-activated (4 days) WT and Hvcn1-deficient CD8^+^ T cells (*n* = 3–4). Quantitative PCR was used to assess expression of mitochondrial genes *16s* and *Nd1* and normalized to the nuclear gene *Hk2* to calculate mitochondrial DNA copy number. Data are presented as mean ± SEM (*n* = 5). Student’s 2-tailed *t* test; *P < 0.05, ***P < 0.005. (**F**) MMP of naive WT and Hvcn1-deficient CD8^+^ T cells was determined using MitoTracker Red (MitoRed). A representative histogram is shown on the right-hand side. Bar charts show mean MFI ± SD (*n* = 3). Data are presented as mean ± SD (*n* = 5). Student’s 2-tailed *t* test; ***P < 0.005.

The severe oxygen consumption rate (OCR) impairment in naive Hvcn1-deficient CD8^+^ T cells led us to focus on this subset for the rest of the study. Preserved CD8^+^ T cell proliferation associated with defective TCR signaling, together with the observed effect of pyruvate deprivation and increased ROS levels on Hvcn1-deficient CD8^+^ T cell division, pointed our attention toward the mitochondria, a primary source of intracellular ROS. We first analyzed the intracellular localization of Hvcn1 in fractionated intracellular components of T cells. We found that in both CD4^+^ and CD8^+^ T cells, a part of intracellular Hvcn1 is present, in addition to the cell surface (Figure 1C), in the mitochondria and at relatively high levels in the CD8^+^ subset (Figure 5, A and B). This localization suggests that Hvcn1 might regulate the flux of protons required to couple the electron transport chain (ETC) with ATP production, particularly in CD8^+^ T cells. Hence, we tested the effect of FCCP [Carbonyl cyanide 4-(trifluoromethoxy) phenylhydrazone] treatment on ROS production by WT and Hvcn1-deficient T cells. FCCP is a mitochondrial uncoupler, which allows protons to diffuse across the mitochondrial membrane and, thus, increase oxygen consumption. As shown in Figure 5C, FCCP treatment increased the proportion of DHE-oxidizing WT, but not Hvcn1-deficient T cells, suggesting that Hvcn1 functions by coupling the ETC with ATP production in T cells.

To further define the role of mitochondrial Hvcn1, we assessed the ratio of mitochondrial and nuclear (M/N) DNA in WT and Hvcn1-deficient T cells as a measure of mitochondrial mass (33).

Compared with their WT counterpart, Hvcn1-deficient naive CD8^+^ T cells displayed significantly reduced M/N DNA (Figure 5, D and E). Upon activation, the M/N DNA became similar in WT and Hvcn1-deficient CD8^+^ T cells.

To further define the role of mitochondrial Hvcn1, we assessed mitochondrial function in naive WT and Hvcn1 T cells by measuring mitochondrial membrane potential (MMP) using MitoTracker Red (MitoRed). As shown in Figure 5F, MMP was significantly increased in Hvcn1-deficient compared with WT-naive CD8^+^ T cells. These data imply that Hvcn1 is required to maintain mitochondrial fitness in naive CD8^+^ T cells.

### Impact of Hvcn1 deficiency on CD8^+^ T cell metabolic switch during activation.

It is well established that activation of naive T cells requires a switch from OXPHOS to aerobic glycolysis (34–36), which produces rapid acidification of the cytosol.

As the major metabolic alterations in Hvcn1-deficient CD8^+^ T cells were observed in the naive T cell subset, we investigated the metabolic configuration of naive and Ab-activated WT and CD8^+^ T cells by combining liquid chromatography-tandem mass spectrometry–based (LC-MS/MS–based) metabolomic and gene expression profiling.

A preliminary analysis of the naive CD8^+^ T cell metabolome revealed that Hvcn1-deficient naive CD8^+^ T cells display an increase in many intermediates of glycolysis as well as of the pentose phosphate pathway (PPP) (Supplemental Figure 6, A and B). Naive Hvcn1-deficient CD8^+^ T cells also display higher levels of TCA cycle intermediates. Given that lactate levels are comparable, the accumulation of glycolysis intermediates may indicate that glucose oxidation is less efficient.

**Figure 6:**
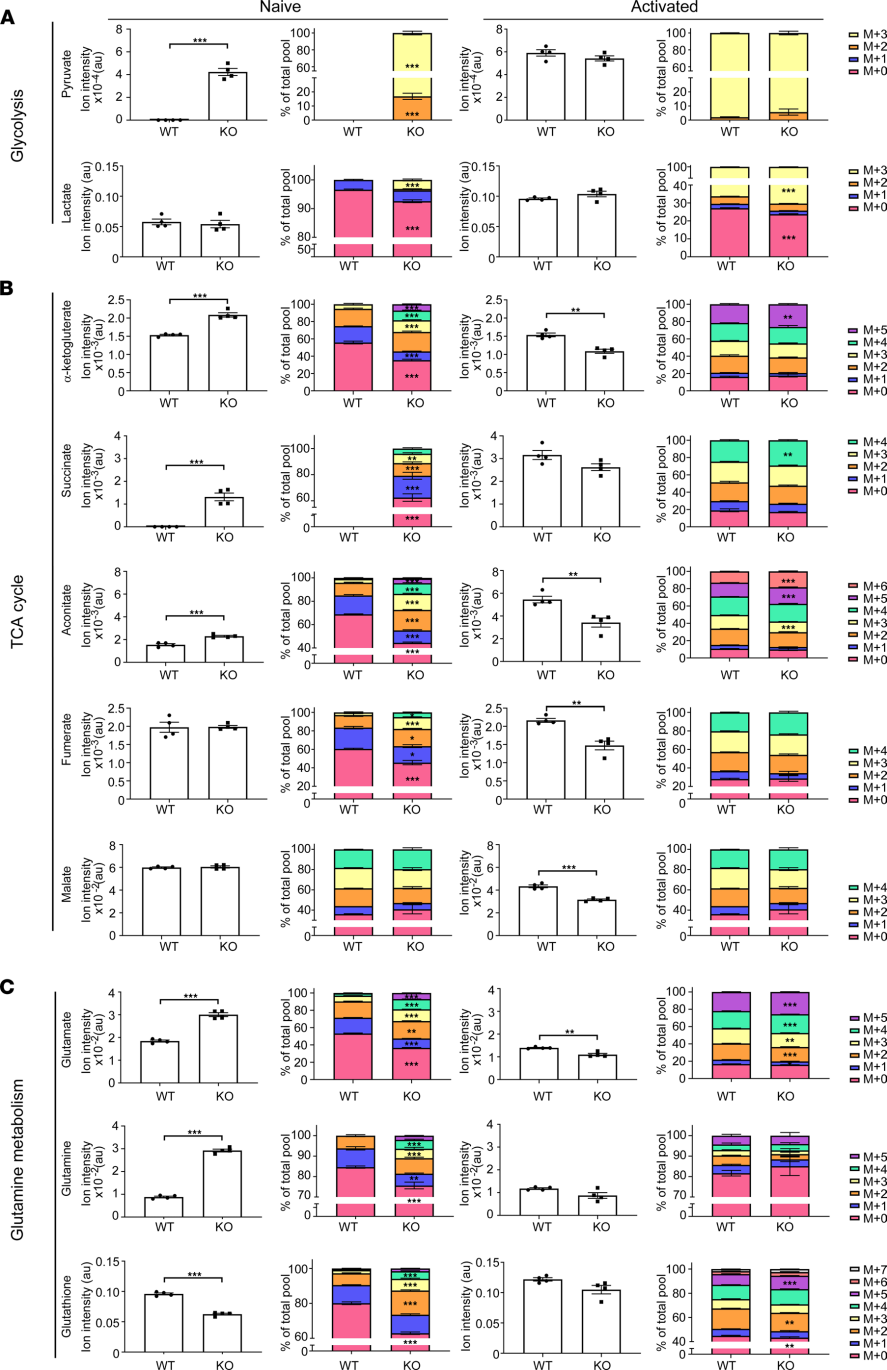
Metabolic analysis of Hvcn1-deficient CD8^+^ T cells. Purified naive and 48-hour activated WT and Hvcn1-deficient CD8 T cells were incubated with _13_C_6_-Glucose for 18 hours, followed by metabolite extraction for LC-MS/MS analysis. Columns 1 and 3 show total levels of each metabolite in the samples. Columns 2 and 4 show the proportion of isotopologues of each metabolite indicated by “M+*n*,” which designates the position in the molecule where the _13_C label is found. (**A**–**C**) Fractional enrichment of glycolysis (**A**), TCA cycle (**B**), and glutamine metabolism (**C**) related _13_C-isotopologues. Data are presented as mean ± SEM; 2-tailed Student’s *t* test; *P < 0.05, **P < 0.01, ***P < 0.001; or Mann-Whitney test. Source data for this figure are available as supplemental material (Supplemental Data).

Based on these observations, we used _13_C_6_-glucose labeling to study metabolic fluxes in naive and activated Hvcn1-deficient CD8^+^ T cells. As shown in Figure 6A, induction of glycolysis was slightly increased as indicated by increased lactate labeling. However, the total level of lactate did not change, in agreement with the extracellular acidification rate (ECAR) data (Figure 4B), where no differences were observed.

Hvcn1-deficient naive CD8^+^ T cells also displayed a more active TCA cycle as suggested by the presence of more metabolites labeled from glucose, including alpha-ketogluterate (α-KG), fumarate, malate, and aconitate, in most cases both via acetyl coenzyme-A (CoA) (m+2 isoform) and pyruvate carboxylation (m+3) (Figure 6B). This compensation might come at the cost of higher ROS production, as we have observed in Hvcn1-deficient CD8^+^ T cells.

Low OCR associated with higher MMP (Figure 4E and Figure 5E) indicates a block at the level of the ATP synthase in the respiratory chain. The higher MMP drives transport of TCA substrates (pyruvate/oxaloacetate) into the TCA cycle, causing more overall labeling, as confirmed by fractional enrichment. The total level of TCA metabolites did not change when comparing Hvcn1-deficient naive CD8^+^ T cells (only the fractions are), except for the increased total level of α-ketoglutarate (α-KG). Increased flux in the TCA cycle is also confirmed by the increased NADH/NAD^+^ ratio (WT: 1.45975211 ± 0.20034345; KO, 1.89728354 ± 0.12170453; *t* test *P* = 0.002). Considering the low OCR, it is likely that NADH is exported from the mitochondria and drives the increased glucose-derived lactate production.

The metabolic defect observed in naive Hvcn1-deficient CD8^+^ T cells is abolished by activation and efficient engagement of the glycolytic flux and TCA cycle (Figure 6, A and B), as reflected by normalization of ECAR and OCR (Figure 4, B–F). Analysis of the TCA cycle flux revealed a lower total level of metabolites in activated Hvcn1-deficient CD8^+^ T cells (succinate, α-KG, fumarate, malate, and aconitate), but no, or only a minor, difference in the fractions (Figure 6B). This could suggest that the way TCA cycle metabolites are produced is the same in WT and activated Hvcn1-deficient CD8^+^ T cells but that possibly some intermediates of TCA cycle are utilized outside the cycle and, thus, overall, they have a lower concentration (e.g., citrate export for lipid synthesis, urea cycle).

### Glutathione is an important antioxidant, which regulates the redox balance of T cells under conditions of ROS accumulation.

In the metabolic flux analysis, we also observed an increase in glutamine and glutamate accompanied by a decrease in glutathione (GSH) in Hvcn1-deficient naive CD8^+^ T cells (Figure 6C). The observed increase in both total level and _13_C_6_-labeled fractions indicated an increased synthesis of these metabolites from glucose. Increased flux of glucose derived carbons was also observed in GSH, but the decreased overall GSH levels indicated a possible consumption/degradation of this metabolite.

### The glutamine/glutamate/GSH pathway was compensated following activation.

Hvcn1-deficient naive CD8^+^ T cells also displayed significantly increased NAD and NADH levels in naive T cells compared with their WT counterpart as well as an increased NAD/NADH ratio (see above and Supplemental Figure 6C). However, the NADP/NADPH ratio was normal in activated Hvcn1-deficient CD8^+^ T cells, which might underlie the restored generation of GSH in these T cells.

While the overall shift in the source of intracellular ATP does not appear to be energetically detrimental as the levels of ATP are maintained, levels of AMP were much higher in Hvcn1-deficient CD8^+^ T cells (Supplemental Figure 6B), suggesting a metabolically stressed status.

Transcription of relevant metabolism genes by CD8^+^ T cells was not affected by lack of Hvcn1 expression (Supplemental Figure 6D).

### AMPK activation contributes to the maintenance of mitochondrial mass in Hvcn1-deficient CD8^+^ T cells.

In conditions of cell stress and nutrient deprivation, AMPK modulates multiple metabolic pathways leading to the inhibition of anabolism to minimize ATP consumption and enhancement of catabolism to stimulate ATP production (37). In addition, AMPK activation results in reduced mTOR signaling, as we have observed during Hvcn1-deficient CD8^+^ T cell activation.

**Figure 7:**
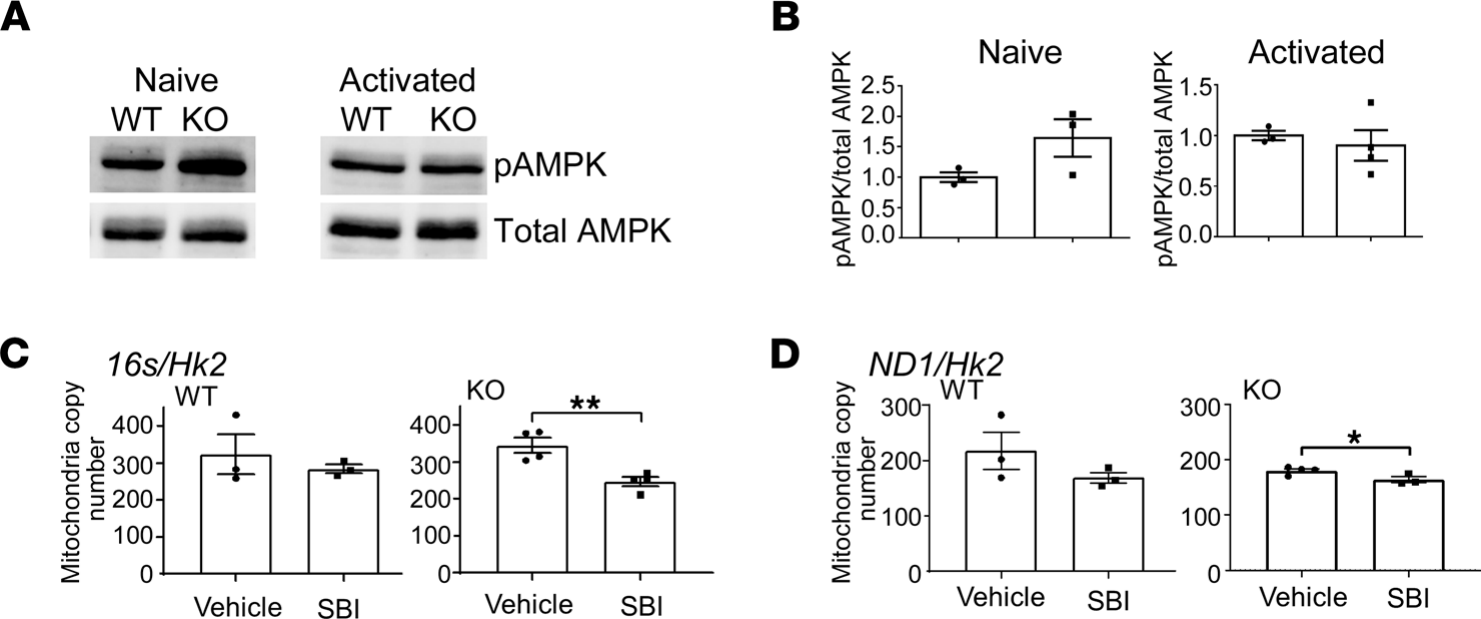
Activation of AMPK maintains mitochondrial mass in Hvcn1-deficient CD8^+^ T cell activation. (**A** and **B**) Purified naive or Ab-activated (4 days) CD8^+^ WT and Hvcn1-deficient T cells were lysed and analyzed by Western blotting for the presence of phosphorylated and total AMPK. Quantification (pAMPK/total AMPK) is shown in **B** (*n* = 3). (**C** and **D**) Total DNA was isolated from Ab-activated (4 days) WT and Hvcn1-deficient CD8^+^ T cells (*n* = 3–4) cultured in the presence of the AMPK inhibitor SBI or vehicle alone. Quantitative PCR was used to assess expression of mitochondrial genes *16s* in **C** and *Nd1* in **D** and normalized to the nuclear gene *Hk2* to calculate mitochondrial DNA copy number. Data are presented as mean ± SEM (*n* > 3). Student’s 2-tailed *t* test; *P < 0.05, **P < 0.01.

Given the increased levels of AMP (Supplemental Figure 6C) associated with mitochondrial dysfunction in Hvcn1-deficient T cells, we measured AMPK activation. In Hvcn1-deficient, but not WT, CD8^+^ T cells, phospho-AMPK (pAMPK) was significantly increased in naive T cells (Figure 7, A and B). AMPK also regulates mitochondrial homeostasis including control of mitochondrial mass through stimulation of mitochondrial biogenesis and mitochondrial fitness through regulation of mitophagy (37). To gain further insights into the role of AMPK activation in Hvcn1-null T cells, we investigated the effect of pharmacologic AMPK inhibition (38) on the mitochondrial dynamics in WT and Hvcn1-deficient T cells following activation. As shown in Figure 7, C and D, pharmacologic inhibition of AMPK activation led to a decreased mitochondrial mass in activated Hvcn1-deficient CD8^+^ T cells, indicating that activated AMPK might contribute to the increase of mitochondrial biogenesis and the normalization of metabolism in activated Hvcn1-deficient T cells.

## Discussion

In this study, we investigated the role of the proton channel Hvcn1 in T cell function. We show that the proton channel Hvcn1 plays a nonredundant role in controlling intracellular acidification in T cells and maintaining mitochondrial fitness in naive CD8^+^ T cells during the transition from naive to activated T cells.

This differs from B cells, in which Hvcn1 functions at the cell membrane by providing ROS for SRC homology 2 domain-containing tyrosine phosphatase (SHP-1) oxidation and subsequent spleen tyrosine kinase (SYK) and AKT activation for BCR signaling. The distinct role of this proton channel in lymphocyte subsets is also suggested by its different expression kinetics and mitochondrial localization in T cells.

Functionally, both Hvcn1-deficient naive CD4^+^ and CD8^+^ T cells share a proliferative defect (more profound in the CD4^+^ subset) and a decreased effector function (cytokine production by CD4^+^ and by CD8^+^ T cells, respectively). These shared features, however, might be the result, at least in part, of a profoundly different metabolic adaptation to intracellular acidification. A cue comes from the recent reports that glycolysis is required for cytokine production by CD4^+^ T cells (39) and that in CD8^+^ T cells, efficient cytolytic activity has been associated with mitochondrial fitness (40).

Despite the proliferative defect, Hvcn1-deficient CD4^+^ T cells display normal ROS production and conserved TCR/CD28 signaling. Reduced proliferation could not be increased by peroxide addition as it was previously described in ubiquinol-cytochrome *c* reductase, Rieske iron-sulfur polypeptide–deficient (Uqcrfs–deficient) T cells (29). Although ROS production is significantly increased in Hvcn1-deficient CD8^+^ T cells, they display impaired AKT/mTOR activation. Collectively, these data suggest that, unlike in B cells, this proton channel is not essential for ROS generation required for antigen-receptor signaling in T lymphocytes.

A fundamental difference between Hvcn1-deficient naive CD4^+^ and CD8^+^ T cells is their susceptibility to cell death upon activation. In particular, a substantial proportion of Hvcn1-deficient CD4^+^ T cells undergo apoptosis, suggestive of an increased sensitivity of CD4^+^ T cells to intracellular acidification. In contrast, CD8^+^ T cell survival appears unaffected by the lack of Hvcn1 expression.

The differential effects of Hvcn1 deficiency in CD4^+^ and CD8^+^ T cells are likely to reflect the distinct metabolic adaptations of the 2 T cell subsets to the increased energy demands imposed by activation in the presence of increased intracellular acidity and mitochondrial dysfunction. The main disturbances appear to affect naive T cells and their metabolic response to activation and appear to be mostly compensated in surviving activated T cells.

In Hvcn1-deficient CD4^+^ T cells, cytokine production is compromised following activation, in line with the blunted glycolytic response (41). The reduced glycolytic rate is likely due to the lower pH (42), as glycolysis-inducing signaling pathways (AKT/mTOR) are preserved. Following activation, surviving Hvcn1-deficient CD4^+^ T cells appear to compensate for this metabolic defect with a transcriptional program aimed at enhancing glycolysis and by shunting TCA cycle intermediates (citrate) to this pathway. Overall, the main role of Hvcn1 in this T cell subset appears to be protection from cell death due to toxic intracellular acidification.

Paradoxically, despite increased ROS production, Hvcn1-deficient CD8^+^ T cells show a defective activation of the AKT/mTORC pathway downstream of the TCR and CD28 signals. This is not due to lack of ROS production, which is increased in Hvcn1-deficient CD8^+^ T cells. The impact of Hvcn1-insufficiency on AKT/mTOR activation in CD8^+^ T cells can be attributed to multiple mechanisms. First, intracellular acidification itself might play a direct role in blunting the mTOR pathway (42), although this effect would also apply to CD4^+^ T cells, in which this pathway appears to be intact. Different from CD4^+^ T cells however, both NAD^+^ and AMP are significantly increased in Hvcn1-deficient CD8^+^ T cells. Both these metabolites are known to activate AMPK and, indeed, significantly higher levels of activated AMPK are detected in Hvcn1-deficient naive CD8^+^ T cells, which, in turn, can blunt mTOR activation. In addition, NAD^+^ might act via Sirtuin 1 (SIRT1), a NAD^+^ dependent deacetylase, which downregulates mTOR signaling in response to nutrients and cellular stress (43).

Compared with their CD4^+^ counterpart, the phenotype of naive CD8^+^ T cells lacking Hvcn1 is dominated by mitochondrial dysfunction. While the glycolytic pathway appears to be relatively unaffected, naive Hvcn1-deficient CD8^+^ T cells have severely impaired mitochondrial respiration and display reduced mitochondrial mass. The increase of mitochondrial polarization might compensate for this defect, leading to an acceleration of the TCA cycle. However, this might come at the cost of higher ROS production (Figure 2D), likely due to the loss of Hvcn1-mediated coupling of ETC and ATP production. As a result, protons are channeled toward ROS production. In addition, GSH production is defective in naive Hvcn1-deficient naive CD8^+^ T cells, possibly contributing to the accumulation of ROS.

These findings raise several questions. First, the nonredundant role of Hvcn1 in naive T cells is surprising, given that, physiologically, these T cells are not exposed to severely acidic compartments. Also, naive T cells mainly rely upon OXPHOS for their survival. It is possible that constitutive OXPHOS metabolism might lead to high proton production, hence, requiring higher removal of protons from both mitochondria and cytoplasm to sustain quiescent naive T cell survival and readiness to respond to function-inducing stimuli. We also show that in vitro-activated Hvcn1-deficient CD8^+^ T cells preferentially differentiate into central memory T cells. The reason for this bias remains unclear. Further, Hvcn1-deficient CD8^+^ T cells significantly upregulate the expression of the inhibitory receptor T cell immunoglobulin and mucin protein 3 but no other markers of T cell senescence. The biological significance of this effect remains to be clarified.

The strength of TCR signals might also play a role in Hvcn1 dependency. A previous study has reported the development of autoimmunity in elderly Hvcn1-deficient mice (21), which might be compatible with the preferential survival of T cells (particularly of the CD4^+^ subset) with lower antigen affinity, which are, therefore, more likely to include autoreactive T cells. However, the Hvcn1-deficient line was followed for 14 months and neither developed spontaneous autoimmunity nor increased in the effector T cell (Teff) subset. The reasons for this discrepancy are unclear but might involve different housing conditions.

Overall, Hvcn1 appears to affect mitochondrial function in naive CD8^+^ T cells, suggesting that this T cell subset relies upon this proton channel to efficiently operate the TCA cycle. To date, no differences have been highlighted in the OXPHOS activity or control of metabolic configuration of different naive T cell subsets prior to activation. Ultimately, our study implies that heterogeneity exists in naive T cell bioenergetics, and this possibility will require more in-depth investigations to be resolved.

Finally, the use of Hvcn1 inhibitors has been suggested as a potential therapeutic approach to malignancies, many of which aberrantly express this proton channel, likely as a mechanism of survival in the highly acidic tumor microenvironment (16, 44). The effect of Hvcn1 on immune competence, as described here and including the studies with one of the most promising Hvcn1 inhibitors (Supplemental Figure 5), suggests that this approach might affect not only the tumor but also the anti-tumor immune response, at least during the priming stage. Therefore, further preclinical studies are needed to validate the viability of this approach in clinical oncology.

## Methods

### Mice.

129P2-*Hvcn1^Gt(RRN293)Byg^*/Mmcd (Hvcn1-deficient; Hvcn1-KO) and WT mice were generated as previously described (20). HY-specific, TCR-transgenic Marilyn, and B6Kd-transgenic mice have been previously described (45, 46).

Recipient mice were depleted of the CD8^+^ T lymphocyte subset by single i.p. injection of 300 μg of anti-CD8 Ab (BioXCell, catalog 2.43) on day –3. On day 0, skin grafting and reconstitution of the CD8^+^ T lymphocyte subset were achieved by adoptively transferring 5 × 10^6^ donor CD8^+^ T cells.

All animal protocols used in this study were approved by the Animal Use and Care Committee of Queen Mary University of London (QMUL), following the Home Office Guidance (Scientific Procedure Act 1986) and the *Guide for the Care and Use of Laboratory Animals* of the National Research Council (National Academies Press, 2011).

### T cell purification and activation.

T cells were purified from the pooled spleen and lymph nodes of WT and Hvcn1-deficient mice using Easy Sep Naive T cell kits or Invitrogen Magnisort naive CD4^+^ or naive CD8^+^ kits as per the manufacturer’s instructions.

T cells were activated with plate-bound anti-CD3 (eBioscience, catalog 17A2) at 1 μg/mL and anti-CD28 (eBioscience, catalog 37.51) at 5 μg/mL with 20 U/mL recombinant human IL-2 (Roche) for 4 days, unless otherwise stated. T cells were activated in complete media containing RPMI 1640 (Thermo Fisher Scientific) supplemented with 50 μM 2-β-Mercaptoethanol (Thermo Fisher Scientific), 2 μM glutamine (Thermo Fisher Scientific), 10 mM HEPES (Thermo Fisher Scientific), 50 IU/mL penicillin (Thermo Fisher Scientific), 50 μg/mL streptomycin (Thermo Fisher Scientific), 1 mM sodium pyruvate (Thermo Fisher Scientific), and 10% FBS (Seralab).

### Generation of BM-derived DCs.

BM-derived DCs were obtained from WT and Hvcn1-deficient mice. Femurs from 8- to 12-week-old mice were removed and BM cells were flushed out with PBS. Red blood cells were lysed. BM cells (5 × 10^6^) were seeded per well in a 6-well plate in RPMI medium supplemented with 10% FCS, 2 mM glutamine, 50 IU/mL penicillin, 50 μg/mL streptomycin, 50 μM 2-ME, and 2% murine GM-CSF obtained from the supernatant of the GM-CSF hybridoma (gift from Jian-Guo Chai, King’s College, London, United Kingdom). Cells were cultured at 37°C in the presence of 5% CO_2_. DCs were matured overnight with 100 ng/mL LPS and used between 7 and 9 days following isolation.

### T helper cell subset differentiation.

T cells were isolated from WT and Hvcn1-deficient mice, activated by plate bound CD3/CD28 Abs, and differentiated toward Th0, Th1, and Th2 phenotypes. Conditions were: Th0 (10 ng/mL IL-2); Th1 (10 ng/mL IL-2; 5 ng/mL IL-12; and 2 μg/mL anti–IL-4); and Th2 (10 ng/mL IL-2; 10 ng/mL IL-4; and 5 μg/mL anti–IFN-γ). All Abs and cytokines were purchased from PeproTech.

### Flow cytometry.

Cells were analyzed for the expression of cell surface markers, intracellular cytokines and transcription factors, markers of proliferation, and viability dyes using a FACS Fortessa (BD) and FlowJo analysis software.

Ex vivo cells were made into single-cell suspensions from the lymph nodes, spleen, and thymus and washed once in PBS (Sigma) with 2% FCS and 2 mM EDTA (Sigma). In vitro–activated cells were harvested and washed in PBS with 2% FCS and 2 mM EDTA. Isolated cells were pelleted and resuspended in 50–100 μL of PBS containing the following Abs: anti–CD3-AF700 (BioLegend, catalog 500A2), anti–CD4-PECF594 (BD, catalog RM4-5), anti–CD8-APCef780 (eBioscience, catalog 53-6.7), anti–CD44-ef450 (eBioscience, catalog IM-7), anti–CD62L-BV605 (BioLegend, catalog MEL-14), anti–LFA-1–PE, anti–CXCR3-PE (eBioscience, catalog CXCR3-173), anti–CCR7-APC (eBioscience, catalog 4B12), anti–CCR4-PE (BioLegend, catalog 2G12), anti–CCR5-PE (eBioscience, catalog HM-CCR5-7A4), anti–GLUT-1 (Rabbit Polyclonal, Novus Bio), anti–CD25–FIT-CELL (BioLegend, catalog PC61), and Aqua (Thermo Fisher Scientific). The Abs anti–mouse IFN-γ PerCP-Cy5.5 (505822), anti–mouse IL-4 PE (504103), anti–mouse T-bet BV421 (644815), anti–mouse PD-1 FITC (135213), anti–mouse CTLA-4 APC106309, anti–mouse LAG-3 PerCP-Cy5.5 (125211), anti–mouse Tim-3 PE/Dazzle 594 (134013), anti–mouse CD11c FITC (117305), anti–mouse 80 PE (104707), anti–mouse 86 BV605 (105125), and anti–mouse I-A^b^ APC (116417) were all from BioLegend.

For intracellular staining, cells were fixed and permeabilized using the eBioscience FoxP3/Transcription Factor Staining Buffer Set for the analysis of transcription factors and BD Cytofix/Cytoperm for the analysis of cytokines.

After Fix/Perm cells were stained with anti–IFN-γ–APC (eBioscience, catalog XMG1.2), anti–IL-2–af488 (eBioscience, catalog JES6-5H4), anti–Granzyme B–FITC-CELL (BioLegend, catalog GB11), anti–Hvcn1 (Alomone, catalog AHC-001), anti–T-bet–BV421 (BioLegend, catalog 4B10), anti–IL-17–ef450 (eBioscience, catalog eBio17B7), and anti–FoxP3-APC (eBioscience, catalog FJK-16s).

Intracellular cytokine staining was performed after a 4-hour PMA and ionomycin stimulation with Brefeldin A (BioLegend, catalog 423304) following surface marker staining, fixation, and permeabilization according to manufacturer’s instructions (BD Biosciences, catalog 554714).

To identify H-2D^b^ HY Uty antigen-specific CD8^+^ T cells in grafted mice, isolated cells were incubated with 3 μL/sample H-2D^b^ Uty Tetramer-PE (MBL) in 100 μL PBS containing 2% FCS for 30 minutes at 37°C with the required cell surface Abs.

To test viability after activation, T cells were labeled with recombinant Annexin V–AF488 and Propidium Iodide using a Dead Cell Apoptosis Kit with Alexa Fluor 488 Annexin V (Thermo Fisher Scientific) as well as FACS Abs for T cell markers as described.

To assess intracellular signaling, fixed and permeabilized T cells were stained with primary Abs against phospho-AKT (Cell Signaling, catalog 193H12), phospho-S6 (Polyclonal, Cell Signaling), phospho-Zap-70 (Cell Signaling, catalog 65e4), and secondary donkey anti–rabbit-AF488 (Polyclonal, Life Technologies).

To measure ROS production, purified T cells were incubated with 5 μM DHE or 2 μM CM-H2DCFDA (Thermo Fisher Scientific) before being activated by streptavidin crosslinked 1 μg/mL anti–CD3-Biotin and 5 μg/mL anti–CD28-Biotin for the indicated time points.

For the pH^i^ assay, naive or day-4 activated T cells were incubated with pHrodo Green AM Intracellular pH Indicator (Life Technologies) as per the manufacturer’s instructions. To calculate the pH^i^ from the MFI, 3 control solutions at pH 5.5, pH 6.5, and pH 7.5 were used to create a standard curve, which was used to calculate the relative pH in the unknown samples.

MMP in naive WT and Hvcn1-deficient T cells was measured using MitoTracker Red CMXRos (150 nM) (Invitrogen).

For the glucose uptake assay, purified naive and activated T cells were resuspended in glucose-free medium containing FACS Abs and incubated with 30 μM 6-NBDG (Thermo Fisher Scientific) for 20 minutes at room temperature.

Samples were acquired on an LSRII Fortessa flow cytometer (BD Biosciences) equipped with 405, 488, 560, and 641 nm lasers; BD cytometer setup and tracking beads were routinely used to calibrate the cytometer. Single stain and fluorescence minus 1 controls were acquired for compensation and precise gating, respectively. Compensation was automatically calculated, and samples were analyzed on dedicated software (FlowJo version 10, FlowJo).

### TUNEL assay.

To assess the cytotoxic ability of CD8^+^ T cells, end-labeling of exposed 3′-OH ends of DNA fragments was undertaken with TUNEL in situ cell-death detection kit fluorescein labeling (Roche Diagnostics) as described by the manufacturer. Briefly, cells were fixed with 4% paraformaldehyde for 1 hour and permeabilized with 0.2% Triton X-100 in PBS for 10 minutes before incubation in the TUNEL reaction mixture. Subsequently, cells were stained with DAPI and visualized under a fluorescence microscope with a 40× objective, and 8 representative areas were randomly selected. At least 500 DAPI^+^ cells were scored. The percentage of apoptotic cells was determined by dividing the number of TUNEL^+^ cells by the total number of DAPI^+^ cells in the corresponding area.

### In vivo cytotoxicity assay.

Splenocytes from female or male WT mice were stained with high (4 μM) and low (2 μM) concentrations of CFSE, respectively, before being injected in primed recipient mice. The proportion of CFSE high to CFSE low was calculated by flow cytometry 1 day later.

### Microscopy.

Purified Hvcn1-deficient and WT CD4 and CD8 T cells were isolated as previously described. Either naive T cells were used on the day of isolation or activated T cells were used in vitro as previously described. Live cells were resuspended in PBS and stained with anti–CD3-APC (Thermo Fisher Scientific, catalog 17A2) and DAPI (Thermo Fisher Scientific) for 40 minutes at room temperature before being washed then fixed and permeabilized using BD Cytofix/Cytoperm. Permeabilized cells were incubated overnight with anti-Hvcn1 Ab (Alomone, catalog AHC-001). Cells were then washed twice with permeabilization buffer before being incubated with goat anti–rat-AF555 (Polyclonal, Life Technologies), goat anti–mouse-AF555 (Polyclonal, Life Technologies) or LAMP-1–PE (eBioscience, catalog RM134L), and donkey anti–rabbit-AF488 (Polyclonal, Life Technologies) for 40 minutes at room temperature. Cells were then washed and attached to a polysine slide before being sealed with Prolong Gold anti-Fade (Life Technologies). Cells were visualized using a Zeiss LSM 710 confocal microscope. Images were analyzed using AxioVision Software.

### Proliferation assays.

For the thymidine proliferation assay, purified T cells were activated as previously described. On day 3, 0.5 μCi/well of tritiated thymidine (New England Nuclear) was added. After 16 hours, cells were harvested onto a filter mat and analyzed using a Wallac Trilux 1450 Microbeta liquid scintillation and luminescence counter (PerkinElmer). The results were presented as cpm.

For the calculation of the mitotic index, T cells were purified and stained with either 4 μM of CFSE (Invitrogen) or 5 μM of Cell Trace Violet (Life Technologies), as per the manufacturer’s instructions, and activated as previously described.

Cells were then harvested and counted on the indicated day. If required, the cells were stained for additional markers for FACS as previously described. The mitotic index was calculated as follows:

The absolute number of T cells in each peak was calculated from the total number of T cells harvested multiplied by the % of T cells in each gate and each peak (t). Each peak was given a division number: 0 for undivided cells, 1 for cells that had undergone 1 division, etc. (D). The number of precursors (P) required for the number of daughters in each peak was P = t/2^D^. The number of mitotic events (Ms) required for the cells in each peak (M) = t-P

The sum of the number of Ms required for each peak equals the total number of Ms.

### Treg suppression assay.

Tregs were purified from WT or Hvcn1-deficient mice using a Treg purification kit (Stem Cell). Tregs were cultured in decreasing ratios with Dynabeads (Invitrogen) purified whole WT T cells labeled with Cell Trace Violet (Thermo Fisher Scientific) for 4 days. The percentage of divided T cells was presented as a readout of suppression.

### Antigen presentation assay.

TCR-transgenic, HY-Dby antigen-specific Marilyn T cells were stained with Cell Trace Violet and cultured with Ficoll (GE HealthCare) purified cells from whole spleen and lymph nodes from male Hvcn1-deficient and WT mice for 4 days. Cells were harvested, stained for surface markers, and analyzed for proliferation.

### Evaluation of mitochondrial mass.

CD8^+^ T cells were isolated from WT and Hvcn1-deficient mice and activated for 4 days as described above. Total DNA was isolated using DNeasy blood and tissue isolation kit (Qiagen, catalog 69504) and eluted in 100 μL of EA buffer. DNA concentration was quantified using NanoDrop and diluted to a final concentration of 10 ng/μL in ddH_2_O. Mitochondria quantification was performed as previously described (33). Briefly, each quantitative PCR (qPCR) reaction was performed using 20 ng of DNA (2 μL) with 5 μL Sybr Green Master Mix (Bio-Rad), 0.5 μL each of forward and reverse primers, and 2 μL of ddH_2_O. The PCR reaction was performed using a Bio-Rad CFX96 thermocycler, at 95°C for 5 minutes followed by 45 cycles at 95°C for 10 seconds, 60°C for 10 seconds, and 72°C for 20 seconds. The mitochondrial copy number was calculated using the equation below:

ΔCt = Ct(nDNA gene) − Ct(mtDNA gene)

Copies of mtDNA = 2 × 2^ΔCt^

### Western blotting.

Total cell lysates were created in Cell Lytic M (Sigma). Isolation of subcellular fractions was performed using the appropriate Isolation Kit for Cultured Cells (Thermo Fisher Scientific). A Bradford assay (Bio-Rad) was used to determine the concentration of protein in each sample, and 20 μg of sample was loaded onto a 12% polyacrylamide-SDS gel (Bio-Rad). Proteins were separated at 100 V, 26 mA for 1 hour. Proteins were transferred to Amersham Protran 0.2 μm nitrocellulose membranes (GE HealthCare) and the membranes were blocked with a 4% milk or 5% BSA solution. Then, 2 μg/mL of primary Abs, anti-Hvcn1, or anti–β-actin (Cell Signaling, catalog 13E5) or anti-GAPDH (Cell Signaling Technology, catalog 5174), used at 1:1000, anti-Hsp60, (Cell Signaling Technology, catalog 12165), used at 1:1000, rabbit anti-NADPH oxidase Ab (1:2,500; Abcam, catalog ab129068), rabbit anti–p-AMPKα (Thr172), and anti-AMPKα (Cell Signaling, D79.5E, catalog 4188 and catalog 2532, respectively) were added to 4 mL of TBS-T with 4% milk or 5 mL of TBS-T with 5% BSA as directed by the manufacturer and incubated for 18 hours at 4°C. Membranes were washed 3 times for 10 minutes each in TBS-T and then incubated with 2 μg/mL donkey anti-rabbit HRP-conjugated secondary Ab (GE HealthCare) for 45 minutes at room temperature. Membranes were washed 5 times in TBS, and ECL reagent (GE HealthCare) was added. Stained protein bands were visualized on photographic film (Fuji) or Chemidoc Imaging system (Bio-Rad).

### In vitro and in vivo migration assays.

Single T cell suspensions were washed twice in complete medium and then counted, after which 0.7 mL of complete medium was added to a 24-well plate with or without 300 ng/mL of CCL21, CCL19, or CXCL10 (Peprotech). A transwell insert (Corning) was added to each well, and 5 × 10^5^ cells in 200 μL of complete medium were layered onto the top of the membrane. At various time points, the inserts were removed and the medium below them was re-suspended; 20 μL of suspension was then removed and the cells counted.

For in vivo migration assays, single-cell suspensions were obtained from lymph nodes of WT or Hvcn1-deficient mice. For naive migration assays, cells were washed once with PBS and labeled with 3 μM CFSE or 1.4 μM DDAO (Invitrogen) before being adoptively transferred into recipient mice.

### Skin grafting.

Donor tail skin was removed and cut into 1 cm^2^ sections. Recipient mice were anesthetized using isoflurane (Halocarbon Products). When the mice reached a surgical plane of anesthesia, 0.26 μg of Metacam (Boehringer Ingelheim) was injected s.c. The mice were then stretched out to ensure easy access to the surgical site using elastic bands. The selected area was shaved, washed with 70% alcohol, and sprayed with Opsite (Smith and Nephew) spray dressing. A piece of skin was removed from the right flank to create a graft bed, any excess blood was removed, and a 1 cm^2^ tail skin graft was placed in the graft bed. The graft was covered with Jellonet (Smith and Nephew). A plaster was then wrapped around the midriff and graft. Mice were put in a warming chamber at 32°C for the plaster to dry and for the mice to recover. Plasters were removed 7–10 days after grafting, and grafts were inspected every other day for signs of rejection. Grafts were considered rejected when less than 10% of the initial tissues remained.

In some experiments, recipient mice were depleted of CD8^+^ T cells by a single i.p. injection of 300 μg of anti-CD8 Ab (BioXCell, catalog 2.43) on day –3. On day 0 (skin grafting), mice received 5 × 10^6^ WT CD8^+^ T cells i.v.

### Metabolic assays.

To analyze the metabolic profile of T cells, we used the Seahorse metabolic analyzer (Agilent) to measure 2 key parameters. The ECAR is a direct measurement of the amount of lactic acid produced and, thus, of glycolysis, whereas the OCR is a measure of OXPHOS. To analyze the glycolytic pathway, T cells were cultured in a glucose-free medium before basal ECAR measurements were taken and then again after the addition of glucose, oligomycin, and 2-deoxy-glucose. To analyze mitochondrial metabolism, we measured the basal OCR and then again after the addition of oligomycin, FCCP, rotenone, and antimycin. We used the WAVE software (Agilent) and report generators to calculate the resulting metabolic parameters. Purified naive or activated T cells were resuspended in 80 μL supplemented XF Base medium (Agilent) at pH 7.4 and added to an XF tissue culture plate, coated with 50 μg/mL poly-d-lysine (Sigma). Cells were quickly spun down, and attachment was confirmed before a further 100 μL of XF Base medium was added. Cells were left to equilibrate for 1 hour at 37°C with no additional CO_2_. The XF-96 sensor cartridge (Agilent) was prepared according to the manufacturer’s instructions before the injection ports were filled with the required reagents.

For the glycolytic stress test, T cells were kept in a base medium with 2 mM glutamine. A final concentration of 10 mM glucose (Sigma), 1 μM oligomycin (Sigma), and 50 mM 2-deoxy-glucose (Sigma) was added as indicated during the test.

For the mitochondrial stress test, T cells were kept in a base medium with 10 mM glucose, 1 mM sodium pyruvate, and 2 mM glutamine. A final concentration of 1 μM oligomycin (Sigma), 1 μM FCCP (Sigma), and 0.5 μM rotenone (Sigma), and antimycin (Sigma) was added as indicated during the test.

### Metabolite extraction and LC-MS/MS analysis.

Cells were harvested in ice-cold PBS, and pellets were resuspended in 250 μL of methanol/acetonitrile 1:1 containing [U-^13^C_6_]-Glucose 1 ng/μL (internal standard, Sigma, 389374) and lysed by TissueLyser for 3 minutes at the highest frequency. Lysates were spun at 20,000*g* for 5 minutes at 4°C. Supernatants were then passed through a regenerated cellulose filter, dried, and resuspended in 100 μL of MeOH for subsequent analysis. Metabolomic data were obtained using an API-4000 triple quadrupole mass spectrometer (AB Sciex) coupled with an HPLC system (Agilent) and autosampler (PAL System). The identity of all metabolites was confirmed using pure standards. Quantification of different metabolites was performed with a LC-MS/MS method using a cyano-phase LUNA column (50 mm × 4.6 mm, 5 μm; Phenomenex). Methanolic samples were analyzed by a 5-minute run in negative ion mode with 30 MRM transitions. The mobile phase A was 5 mM ammonium acetate pH 7.00 in MeOH. The gradient was 100% A for all the analysis with a flow rate of 500 μL/min. MultiQuant software (version 3.0.2) was used for data analysis and peak review of chromatograms. Quantitative evaluation of all metabolites was performed based on calibration curves with pure standards, and then data were normalized on micrograms of proteins analyzed by the Bradford method.

### Metabolic labeling and metabolome analysis.

Naive CD8^+^ T cells were isolated from WT and Hvcn1-deficient mice using a MojoSort mouse isolation kit (BioLegend, catalog 480044) following manufacturer’s instructions. Naive cells were cultured in glucose-free RPMI (Thermo Fisher Scientific, catalog 11879020) supplemented with 11.1 mM _13_C_6_-glucose (Cambridge Isotope Laboratories, catalog CLM-1396-5) and HEPES (_13_C-G media) for 18 hours. This time point was determined by pilot studies. For activated cells, isolated CD8^+^ T cells were cultured in T cell culture media for 48 hours on CD3/CD28 coated plates to activate the cells. The cell media was then changed to _13_C-G media and cultured for an additional 18 hours. After culture, both naive and activated cells were processed in a similar manner to isolate metabolites. Cells were washed with PBS 3 times and resuspended in ice cold extraction buffer (50% methanol, 30% acetonitrile, 20% ultrapure water, and 100 ng/mL HEPES) at a ratio of 20 × 10^6^ cells/mL. Cells were incubated on methanol and dry ice for 15 minutes and then placed on a shaker for 15 minutes at 4°C, followed by incubation at –20°C for 1 hour. Cell lysate was centrifuged, and the supernatant collected and transferred to autosampler glass vials which were stored at –80°C until further analysis.

LC-MS/MS analysis was performed using a Q Exactive Quadrupole-Orbitrap mass spectrometer coupled to a Vanquish UHPLC system (Thermo Fisher Scientific). The liquid chromatography system was fitted with a Sequant ZIC-pHILIC column (150 mm × 2.1 mm) and guard column (20 mm × 2.1 mm) from Merck Millipore and the temperature maintained at 35°C. The sample (2 μL) was separated at a flow rate of 0.1 mL/min. The mobile phase was composed of 10 mM ammonium carbonate and 0.15% ammonium hydroxide in water (solvent A) and acetonitrile (solvent B). A linear gradient was applied by increasing the concentration of A from 20% to 80% within 22 minutes and then maintained for 7 minutes. The mass spectrometer was operated in full MS and polarity switching mode, in the range of 70–1000 *m/z* and resolution 70,000. Major ESI source settings were: spray voltage 3.5 kV, capillary temperature 275°C, sheath gas 35, auxiliary gas 5, AGC target 3e6, and maximum injection time 200 ms. For the targeted analysis, the acquired spectra were analyzed using XCalibur Qual Browser and XCalibur Quan Browser software (Thermo Fisher Scientific). Compound discoverer 3.1 (Thermo Fisher Scientific) was used for untargeted and potentially novel feature detection and annotation with library scoring. Features with the fold change greater than 2 and *P* < 0.05 were selected as discriminating markers.

Samples were analyzed by quadruplicate.

### AMP kinase inhibition.

Purified naive T cells were Ab-activated in the presence of the selective AMP-kinase inhibitor SBI-0206965 (38) (2 μM; Cell Signaling, catalog 29089) or DMSO vehicle control for 4 days prior to analysis.

### Quantitative PCR.

For the analysis of gene transcripts, mRNA was isolated from purified naive or activated T cells using Qiagen RNeasy Mini Kits following the manufacturer’s instructions. cDNA was generated using iScript Reverse Transcription Super mix (Bio-Rad) following the manufacturer’s instructions. A total of 20 μg of cDNA was mixed with a final concentration of 0.5 μM of the forward and reverse primers and the recommended amount of Sybr Green (Bio-Rad) reagents. The mixture was added to a Bio-Rad CFX Connect qPCR machine and run at 95°C for 3 minutes to start, then 40 cycles of 95°C for 10 seconds, and 55°C for 30 seconds. The results were analyzed using the ΔΔCt method. The primers used are listed in Supplemental Table 1.

### Statistics.

All data are presented as mean (±SD) unless otherwise stated. Tests of normality were performed on all data. Standard paired and unpaired *t* tests were performed on normally distributed data and, if comparisons were made between more than 2 groups, a 1-way ANOVA test was performed. If the 1-way ANOVA was statistically significant, a Tukey’s post hoc test for multiple comparisons was used. All nonparametric data were assessed with Wilcoxon’s signed rank and Mann-Whitney *U* tests for paired and unpaired data, as appropriate.

Statistical analysis was performed using Prism (Version 8.3.0, GraphPad Software). A 2-tailed *P* value of less than 0.05 was considered statistically significant with all tests performed.

### Study approval.

All animal protocols used in this study were approved by the Animal Use and Care Committee of QMUL, following the Home Office Guidance (Scientific Procedure Act 1986), and the *Guide for the Care and Use of Laboratory Animals* of the National Research Council (National Academies Press, 2011).

## Author contributions

DC and TP performed and analyzed the data from most experiments. FB, GW, HF, VM, AM, NM, EJW, SN, and KCPC performed experiments and analyzed the data. MC, GDN, and FMMB designed experiments. KB, DA, GDN, MC, and FMMB wrote the manuscript. All authors discussed and revised the manuscript.

## Supplementary Material

Supplemental data

Supplemental data set 1

## Version 1 04/26/2022

In-Press Preview

## Version 2 05/23/2022

Electronic publication

## References

[1] Johnson BA (1995). Mechanisms of myocardial hypercarbic acidosis during cardiac arrest. J Appl Physiol (1985).

[2] LaManna JC (1995). Rapid recovery of rat brain intracellular pH after cardiac arrest and resuscitation. Brain Res.

[3] Damaghi M (2013). pH sensing and regulation in cancer. Front Physiol.

[4] Brand A (2016). LDHA-associated lactic acid production blunts tumor immunosurveillance by T and NK cells. Cell Metab.

[5] Fischer K (2007). Inhibitory effect of tumor cell-derived lactic acid on human T cells. Blood.

[6] Masson D (1990). Interaction of chondroitin sulfate with perforin and granzymes of cytolytic T cells is dependent on pH. Biochemistry.

[7] Mendler AN (2012). Tumor lactic acidosis suppresses CTL function by inhibition of p38 and JNK/c-Jun activation. Int J Cancer.

[8] Calcinotto A (2012). Modulation of microenvironment acidity reverses anergy in human and murine tumor-infiltrating T lymphocytes. Cancer Res.

[9] Casey JR (2010). Sensors and regulators of intracellular pH. Nat Rev Mol Cell Biol.

[10] Ramsey IS (2006). A voltage-gated proton-selective channel lacking the pore domain. Nature.

[11] Sasaki M (2006). A voltage sensor-domain protein is a voltage-gated proton channel. Science.

[12] El Chemaly A (2010). VSOP/Hv1 proton channels sustain calcium entry, neutrophil migration, and superoxide production by limiting cell depolarization and acidification. J Exp Med.

[13] Thomas RC, Meech RW (1982). Hydrogen ion currents and intracellular pH in depolarized voltage-clamped snail neurones. Nature.

[14] Murphy R (2005). Voltage-gated proton channels help regulate pHi in rat alveolar epithelium. Am J Physiol Lung Cell Mol Physiol.

[15] DeCoursey TE (2013). Voltage-gated proton channels: molecular biology, physiology, and pathophysiology of the H(V) family. Physiol Rev.

[16] Seredenina T (2015). Voltage-gated proton channels as novel drug targets: from NADPH oxidase regulation to sperm biology. Antioxid Redox Signal.

[17] Capasso M (2010). HVCN1 modulates BCR signal strength via regulation of BCR-dependent generation of reactive oxygen species. Nat Immunol.

[18] Cherny VV (2003). Properties of single voltage-gated proton channels in human eosinophils estimated by noise analysis and by direct measurement. J Gen Physiol.

[19] DeCoursey TE (2010). Voltage-gated proton channels find their dream job managing the respiratory burst in phagocytes. Physiology (Bethesda).

[20] Ramsey IS (2009). Hv1 proton channels are required for high-level NADPH oxidase-dependent superoxide production during the phagocyte respiratory burst. Proc Natl Acad Sci U S A.

[21] Sasaki M (2013). Autoimmune disorder phenotypes in Hvcn1-deficient mice. Biochem J.

[22] Schilling T (2002). Voltage-activated proton currents in human lymphocytes. J Physiol.

[23] Asuaje A (2017). The inhibition of voltage-gated H^+^ channel (HVCN1) induces acidification of leukemic Jurkat T cells promoting cell death by apoptosis. Pflugers Arch.

[24] Kim DG (2017). Clinical significance of lactate clearance for the development of early allograft dysfunction and short-term prognosis in deceased donor liver transplantation. Clin Transplant.

[25] Chen Y (2003). The male minor transplantation antigen preferentially activates recipient CD4+ T cells through the indirect presentation pathway in vivo. J Immunol.

[26] Valujskikh A (2002). Cross-primed CD8(+) T cells mediate graft rejection via a distinct effector pathway. Nat Immunol.

[27] Rybicka JM (2012). Phagosomal proteolysis in dendritic cells is modulated by NADPH oxidase in a pH-independent manner. EMBO J.

[28] Wolf Y (2020). TIM3 comes of age as an inhibitory receptor. Nat Rev Immunol.

[29] Sena LA (2013). Mitochondria are required for antigen-specific T cell activation through reactive oxygen species signaling. Immunity.

[30] Guarino VA (2019). Reaction rate of pyruvate and hydrogen peroxide: assessing antioxidant capacity of pyruvate under biological conditions. Sci Rep.

[31] Thi Tran U, Kitami T (2019). Niclosamide activates the NLRP3 inflammasome by intracellular acidification and mitochondrial inhibition. Commun Biol.

[32] Hong L (2013). Voltage-sensing domain of voltage-gated proton channel Hv1 shares mechanism of block with pore domains. Neuron.

[33] Quiros PM (2017). Analysis of mtDNA/nDNA ratio in mice. Curr Protec Mouse Biol.

[34] Vander Heiden MG (2009). Understanding the Warburg effect: the metabolic requirements of cell proliferation. Science.

[35] Yang K, Chi H (2012). mTOR and metabolic pathways in T cell quiescence and functional activation. Semin Immunol.

[36] Fox CJ (2005). Fuel feeds function: energy metabolism and the T-cell response. Nat Rev Immunol.

[37] Herzig S, Shaw RJ (2018). AMPK: guardian of metabolism and mitochondrial homeostasis. Nat Rev Mol Cell Biol.

[38] Dite TA (2018). AMP-activated protein kinase selectively inhibited by the type II inhibitor SBI-0206965. J Biol Chem.

[39] Cho SH (2019). Hypoxia-inducible factors in CD4^+^ T cells promote metabolism, switch cytokine secretion, and T cell help in humoral immunity. Proc Natl Acad Sci U S A.

[40] Swadling L (2020). Human liver memory CD8^+^ T cells use autophagy for tissue residence. Cell Rep.

[41] Chang CH, et alet al (2013). Posttranscriptional control of T cell effector function by aerobic glycolysis. Cell.

[42] Balgi AD (2011). Regulation of mTORC1 signaling by pH. PLoS One.

[43] Jeng MY (2018). Metabolic reprogramming of human CD8^+^ memory T cells through loss of SIRT1. J Exp Med.

[44] Fernandez A, et al Pharmacological modulation of proton channel Hv1 in cancer therapy: future perspectives (2016). Mol Pharmacol.

[45] Lantz O (2000). Gamma chain required for naive CD4+ T cell survival but not for antigen proliferation. Nat Immunol.

[46] Tsang JY (2008). Conferring indirect allospecificity on CD4+CD25+ Tregs by TCR gene transfer favors transplantation tolerance in mice. J Clin Invest.

