# Supplementary material for: Loss of voltage-gated hydrogen channel 1 expression reveals heterogeneous metabolic adaptation to intracellular acidification by T cells
Source: JCI Insight. 2022 May 23;7(10):e147814. doi: 10.1172/jci.insight.147814 (PMC9220931; doi:10.1172/jci.insight.147814)
Supplement: Supplemental data [file jciinsight-7-147814-s050.pdf]

Supplemental Figures

Supplemental Figure 1. Cellularity in the thymus and spleen and normal suppressive function of regulatory T-cells.

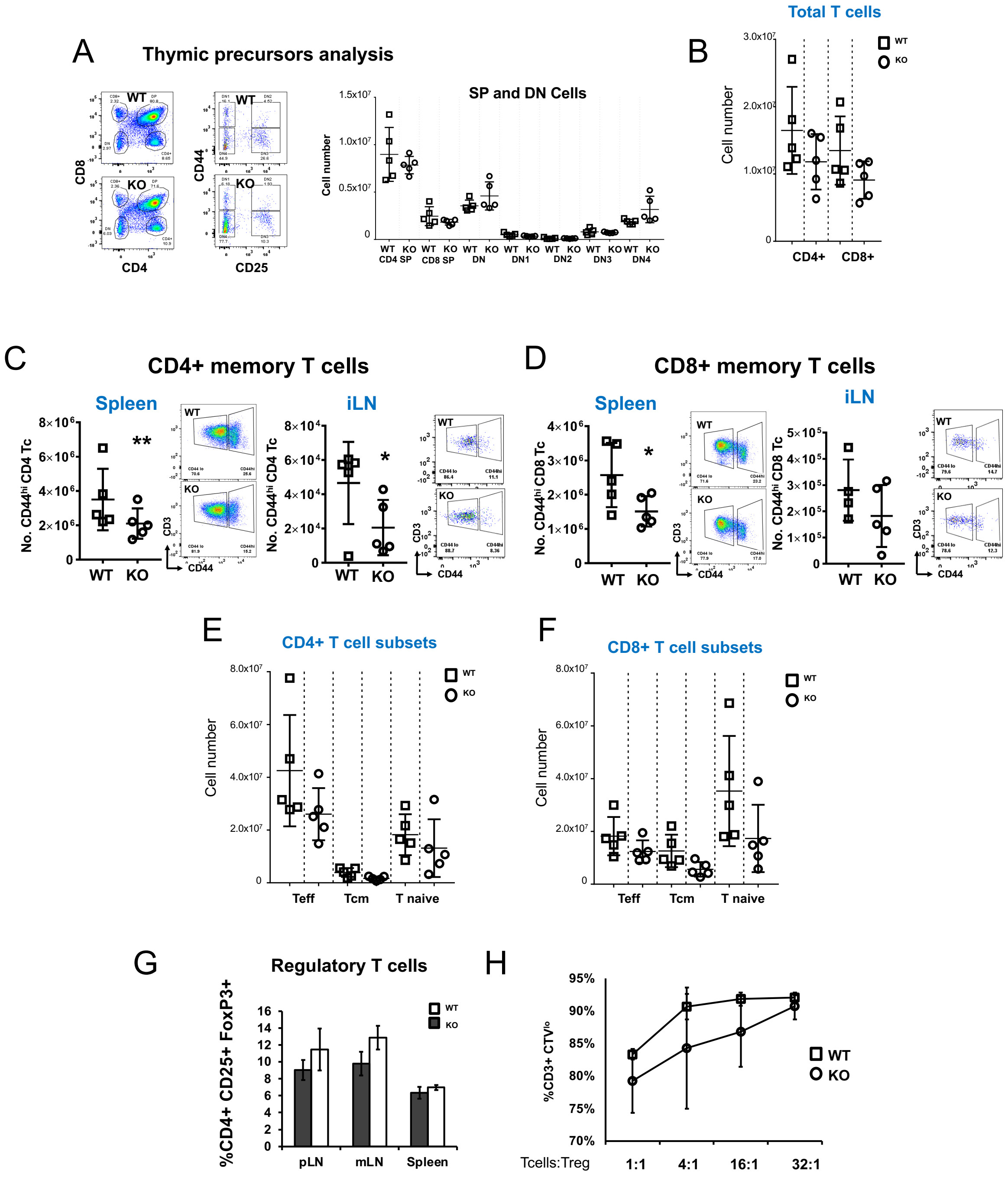

A: Live cells in the thymus from 5 Hvcn1-deficient and 5 WT mice were counted after harvest and then stained, for flow cytometric analysis, with antibodies against CD3,

CD4, CD8, CD25, CD62L and CD44. The proportions of thymic subsets were calculated after gating on singlets and live cells. Representative dot-plots are shown on the right-hand side of the panel. The number of CD4 single positive, CD8 single positive and the varying double negative subtypes was calculated by multiplying the number of cells counted by the proportion in each flow cytometry gate. (+/-SD; n=5).

B-F: T-cells from the spleen and lymph nodes of 5 Hvcn1-deficient and 5 WT mice were stained for flow cytometry, with antibodies against CD3, CD4, CD8, CD25, CD62L and CD44. B: Total CD4+ and CD8+ T cells (pooled spleen and lymph nodes). C-D: CD44high CD4+ and CD8+ T-cells from either spleen or inguinal lymph nodes of WT and Hvcn1-deficient mice were analyzed and counted by flow cytometry. *p<0.05; **p< 0.01; 2-tailed Students T test

E: CD4+ T-cell subsets; F: CD8+ T-cell subsets.

G: T-cells from 5 Hvcn1-deficient and 5 WT mice were stained with antibodies against CD3, CD4, CD25 and FoxP3 and analyzed by flow cytometry.

H: Treg were purified from single cell suspensions of spleen and lymph nodes from WT or Hvcn1-deficient mice. Responder T-cells, from WT C57BL/6 mice were stained with Cell Trace Violet (CTV) and incubated at varying proportions with Treg plus anti-CD3/28 plus 20U/ml IL-2. On day 4 T-cells were stained with anti-CD3 and analyzed by flow cytometry and the percentage of CD3+ CD44high was calculated (+/-SD; n=3).

Supplemental Figure 2. Gating strategy for flow cytometric analysis.

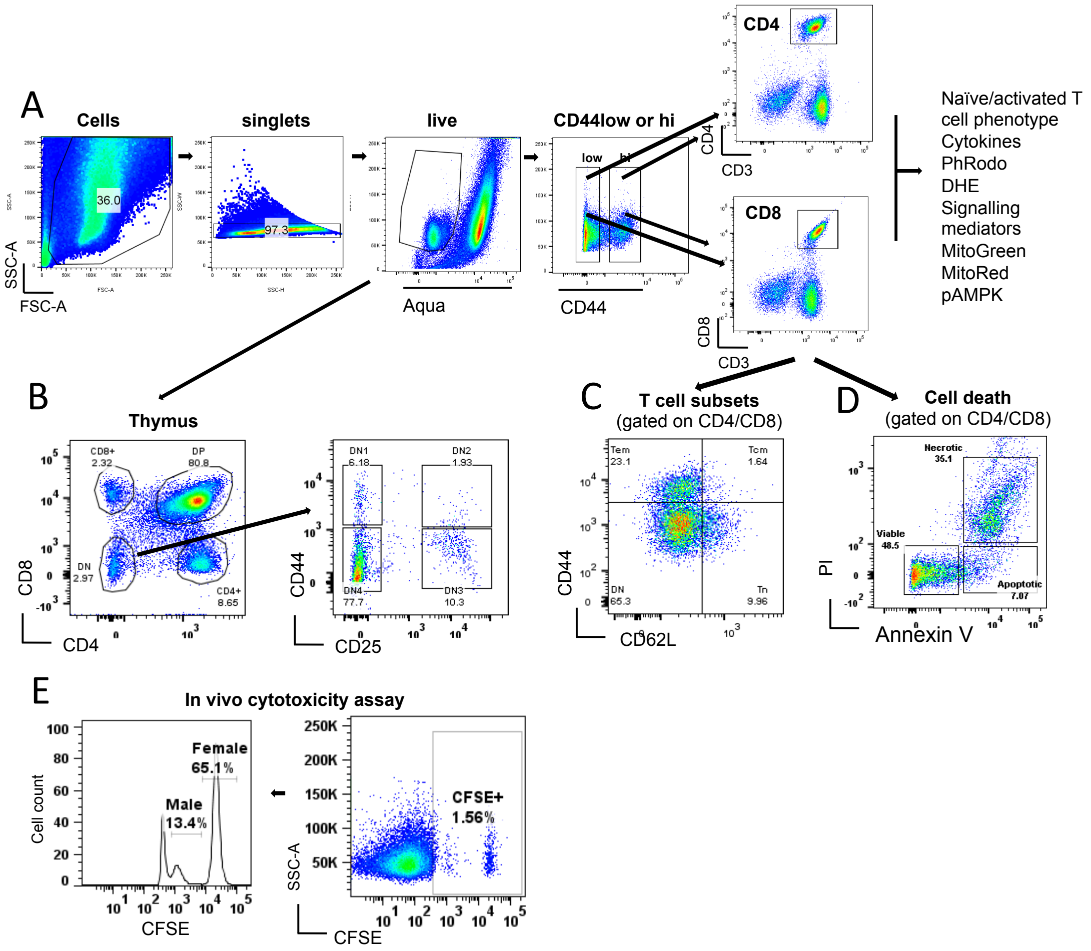

A: Exemplar flow cytometry gating strategy to define singlets, live cells, memory (CD44 high) and naïve (CD44low) in the CD4+ or CD8 T-cell subsets. Once identified, each subset was further analyzed for the expression of the indicated markers.

B: Gating strategy for the identification of thymocyte subpopulations.

C: Gating strategy for the identification of T-cell subsets.

D: Gating strategy for the analysis of T-cell death.

E: Gating strategy for in vivo cytotoxicity assay.

Supplemental Figure 3. Functional features of Hvcn1-deficient T-cells

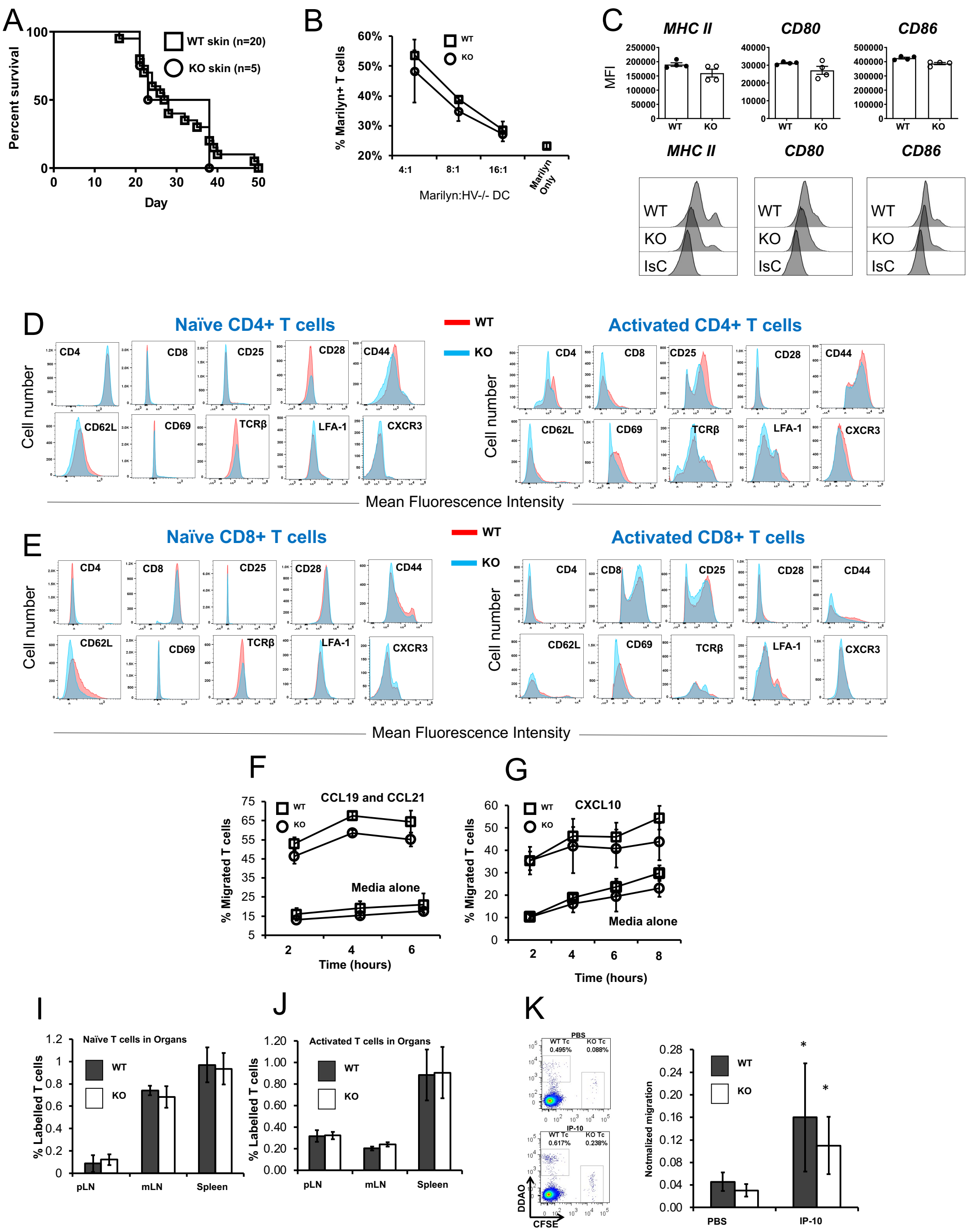

A: Kinetics of graft rejection of WT (n=20) or Hvcn1-deficient (n=5) male skin by female WT mice. Grafts were considered rejected when less than 10% of the initial tissues remained. Results are presented as a survival curve.

B: Single cell suspensions of spleen and lymph nodes from female Thy1.1+ Marilyn mice were stained with Cell Trace Violet (CTV) and incubated with varying ratios of whole spleen from male Hvcn1-deficient (n=3) or WT mice (n=3) for 4 days. Cells were harvested, stained and fixed and the proportion of CD3+, Vβ6+, Thy1.1+ CTV negative T-cells was calculated (+/- SD; n=3).

C: Phenotype of mature WT and Hvcn1-deficient BM-derived DCs. The bar-graphs show the mean MFI of the indicated markers (+/- SD; n=3). Representative histograms are shown below.

D-E: Expression of key molecules on Hvcn1-deficient T-cells. Naive T-cells (left hand side) or 4-day antibody-activated T-cells (right hand side) were purified, stained for cell surface molecules then fixed and permeabilized and stained for intracellular molecules. T-cells were analyzed by flow cytometry and gated on either CD4+ (D) or CD8+ (E) T-cells. Student's T-test *$p<0.05$

F-G: To measure migration *in vitro*, purified Hvcn1-deficient or WT naïve T-cells were added on the top of a transwell© and medium with or without CCL19 (200ng/ml) and CCL21 (200ng/ml) was added to the lower chamber (F). Cells that had migrated to the lower chamber were counted at the indicated time-points and the results are shown as the mean percentage of the original population seeded (+/-SD; n=3). Alternatively, Hvcn1-deficient or WT naïve T-cells were antibody-activated for 4 days, and the same experiment was performed using media with or without CXCL-10 (300ng/ml, H), (+/-SD; n=6).

H-I: To measure migration *in vivo*, purified naïve (H) or antibody-activated (I) Hvcn1-deficient or WT T-cells were stained with CFSE or DDAO and adoptively transferred (10^7) into WT recipients. After 24hr mice were sacrificed and the percentage of each cell type in the indicated lymphoid organs was analyzed by flow cytometry. The graphs show the mean percentage of labeled T-cells retrieved +/- SD; n=3).

J: WT recipient mice were administered with CXCL-10 (300 ng) i.p.. Labeled activated Hvcn1-deficient or WT T-cells were injected i.v. and their localization to the

peritoneal cavity was assessed by flow cytometry. The graphs show the mean migration (normalized for the number of cells injected) of labeled T-cells retrieved in the peritoneal lavage (+/-SD; n=3).

Supplemental Figure 4. Glucose uptake by Hvcn1-deficient T-cells.

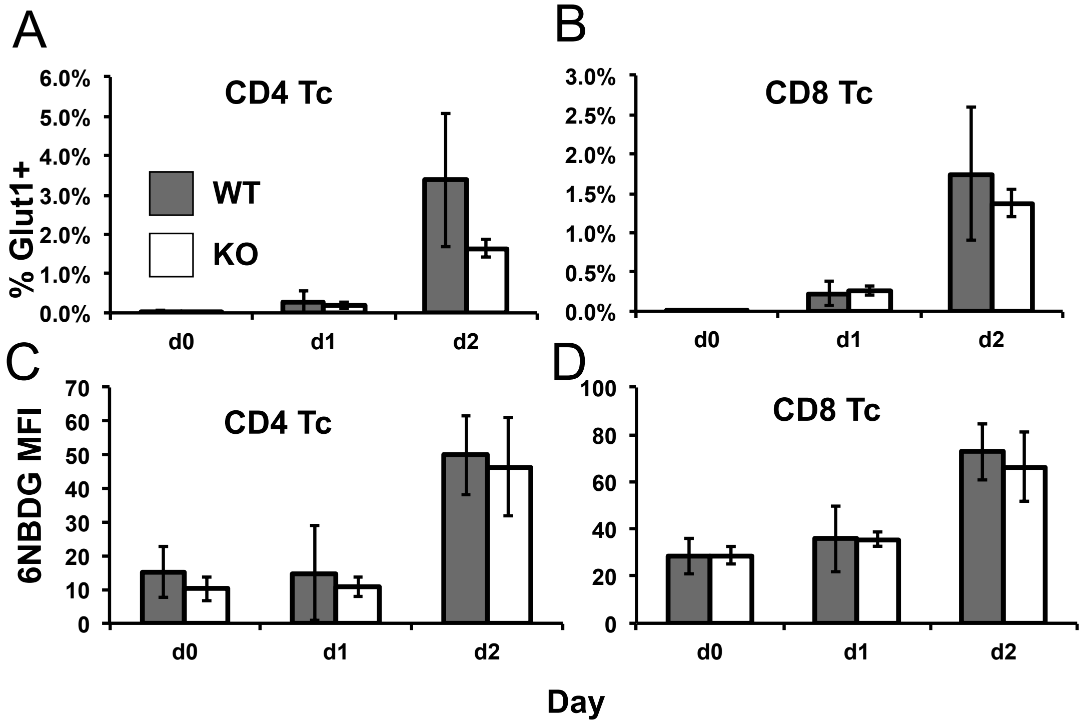

To assess glucose utilization of by Hvcn1-deficient T-cells, purified WT or Hvcn1-deficient T-cells were activated as previously described, for the indicated number of days. Cells were analyzed by FACS for expression of the glucose transporter Glut-1 (A-B) and their ability to take up the fluorescent glucose analogue 6NBDG (C-D) (+/- SD; n=3).

Supplemental Figure 5. The Hvcn1 Inhibitor 2-Guanidinobenzimidazole (2GBI) recapitulates Hvcn1 genetic deletion.

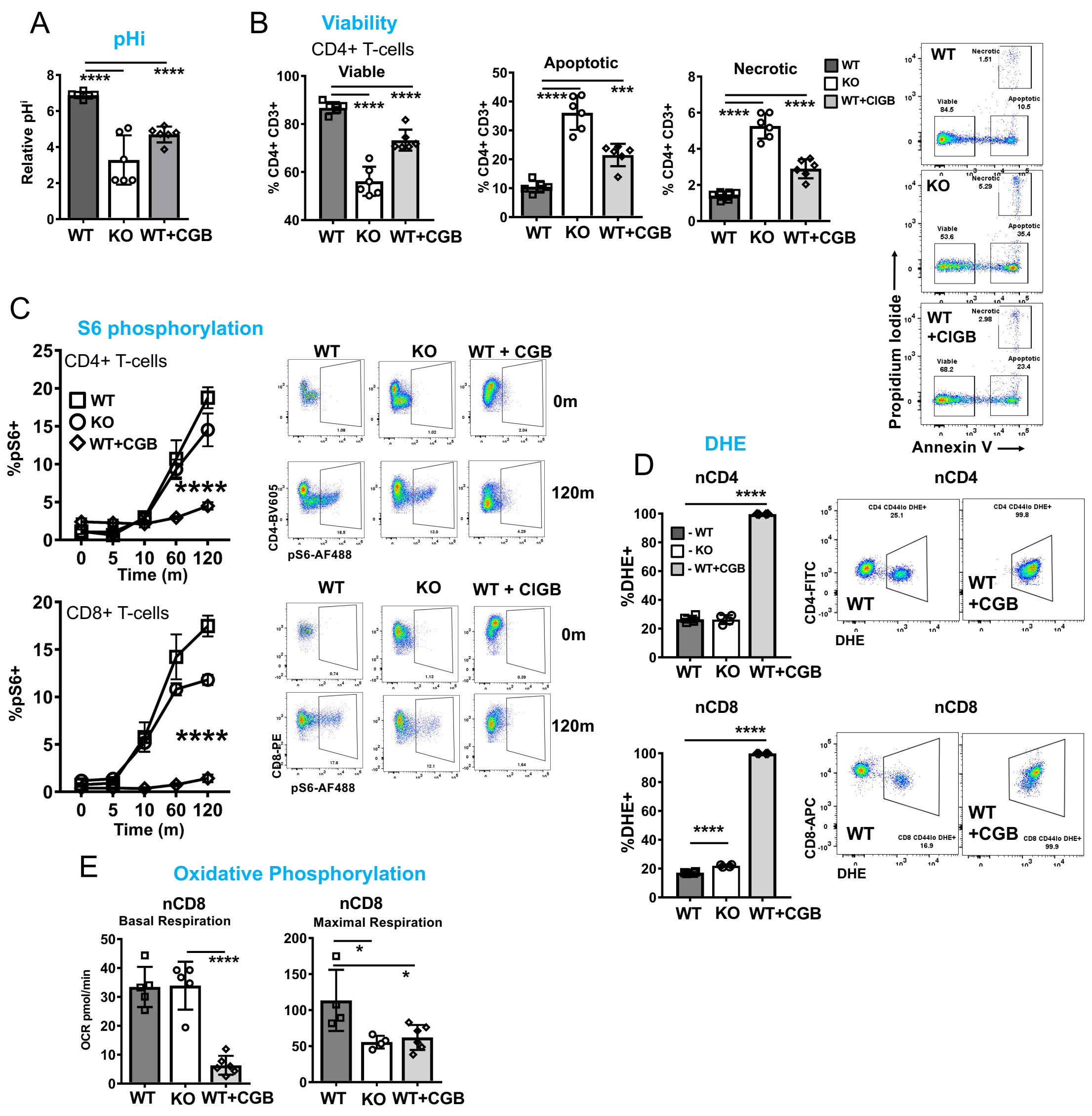

A: Mean pH^{i} of WT nCD8 incubated for 2 hours with or without 200μM 5-Chloro-2-Guanidinobenzimidazole (CGB). For comparison, pH^{i} of Hvcn1-deficient T-cells is shown. Data are shown as bar-charts (+/- SD; n=6) B: Mean percentage of viable, apoptotic and necrotic CD4+ T-cells after antibody activation in the presence of 200μM CGB for 16h are shown (+/- SD; n=6) with representative dot-plots. C: Mean proportion of T-cells with phosphorylation of S6 in nCD4 (top) and nCD8 (bottom)

after activation with or without CGB measured at the indicated time points (+/-SD; n=4) and representative dot-plots. D: Superoxide production by naïve CD4 and CD8 T cells incubated with or without 200μM CGB. Data are shown as mean +/- SD (n=4) and representative dot-plots. E: Basal and maximal respiration of nCD8 incubated with or without 200μM CGB were calculated from the oxygen consumption rate (OCR; pmol/min). Data are shown as mean +/-SD (n=10-12). Two-way ANOVA with Tuckey post-hoc test. *p<0.05; ****p< 0.001

Supplemental Figure 6. Naïve and activated Hvcn1-deficient CD8+ T-cell metabolomics and metabolic gene expression.

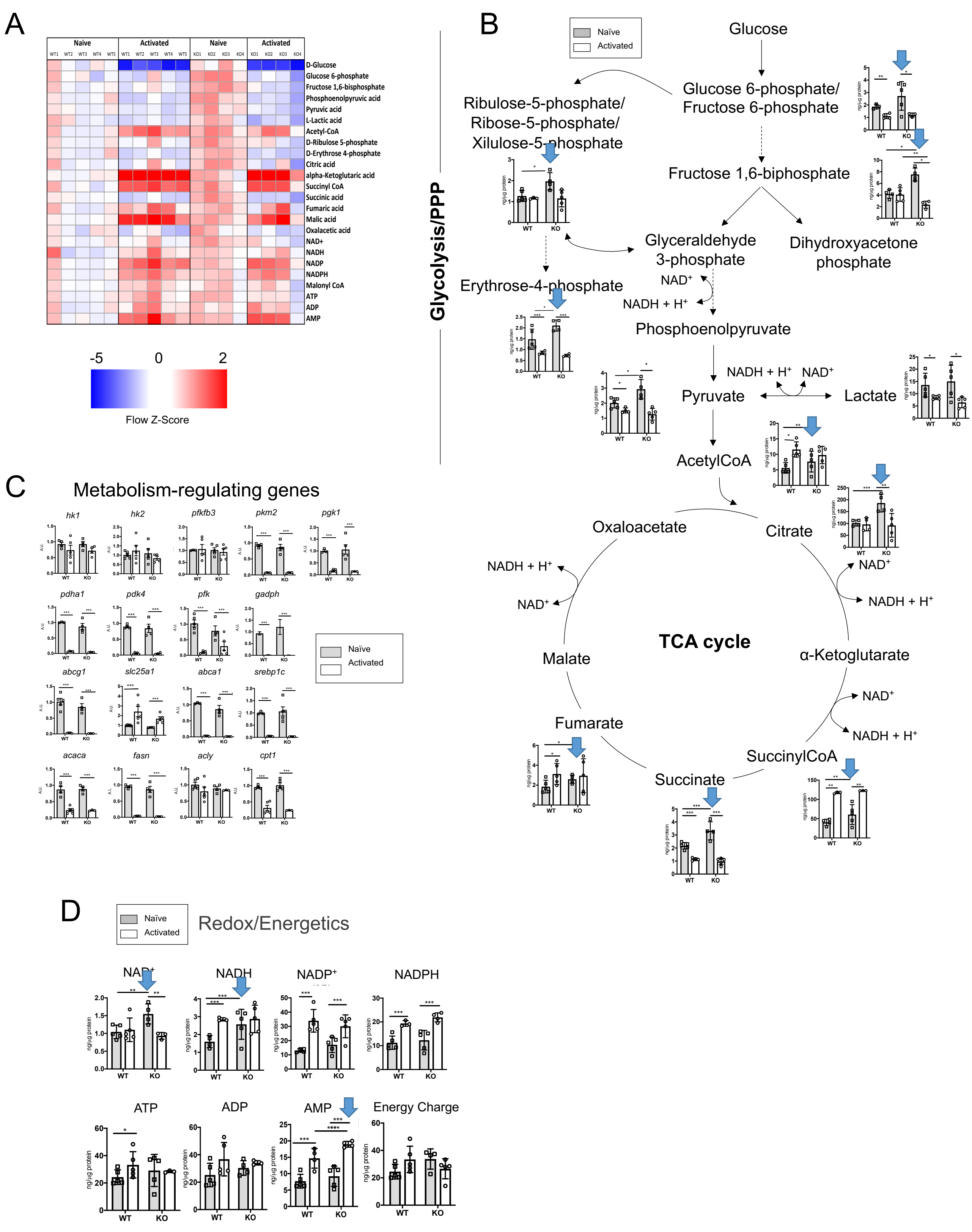

Naive and activated (4 days) WT and Hvcn1-deficient CD8+ T-cells from 5 independent samples underwent metabolomic analysis and metabolic gene expression profiling.

A: Heatmap of metabolites detected in the T-cell populations.

B: The concentration of the indicated intracellular metabolites in naïve and activated CD8+ T-cells was analyzed, and results presented as ng of metabolite per µg of protein (+/-SD; N=5). The metabolites have been grouped into their key pathways.

C: Relative expression of metabolic genes in WT and Hvcn1-deficient naïve CD8+ and activated CD4+ T-cell was calculated by QRT-PCR.

D: Metabolites involved in redox/energetic pathways.

Results are presented as mean +/- SD (N=5). The arrows indicate major metabolic changes. One-way Anova with Tuckey post-hoc test. *p<0.05, ** p<0.01, ***p<0.005, ****p<0.001

Table S1: primers for q-PCR used in this study.

Msrebp-1c	NT_096135	Fwd	ATGGATTGCACATTTGAAGACATGCT
		Rev	CCTGTGTCCCCTGTCTCAC
Macaca (Acc1)	NM_133360	Fwd	AAGGCTATGTGAAGGATGTGG
		Rev	CTGTCTGAAGAGGTTAGGGAAG
Mfasn	NM_007988	Fwd	TCGTGATGAACGTGTACCGG
		Rev	CGGGTGAGGACGTTTACAAAG
Macly	NM_134037	Fwd	CTGACCTTGCTGAACCCC
		Rev	CCCGAGTATTCCCCGTAAT
Mslc25a1	NM 153150	fwd	CAGAAGCAGTGGTAGTCGTG
		rev	TTCCCTTTAGCCCTTGTTCC
Mcpt-1a	NM_013495	fwd	CCAAGTATCTGGCAGTCGA
		rev	CGCCACAGGACACATAGT
Mhk2	NM_013820	fwd	TCAAAGAGAACAAGGGCGAG
		rev	AGGAAGCGGACATCACAATC
Mpkm2	NM_011099	fwd	CCATTCTCTACCGTCCTGTTG
		rev	TCCATGTAAGCGTTGTCCAG
Mpdha1	NM_008810	fwd	ACATGGCTTCACCTTCACTC
		rev	CCGTTGCCTCCATAGAAGTTC
Mpdk4	NM_013743	fwd	AGTGACTCAAAGACGGGAAAC
		rev	GTGTGAGGTTTAATTCTGGCG
Mpfk1	NM_008824	fwd	AACCTTAGAGGCCGCAT
		rev	GAATGGTCCTCTTCATGTGG
Mabca1	NM_013454	fwd	GGTTTGGAGATGGTTATACAATAGTTGT
		rev	CCCGGAAACGCAAGTCC
Mabcg1	NM_009593	fwd	TTCATCGTCCTGGGCATCTT
		rev	CGGATTTTGTATCTGAGGACGAA

Mpfkfb3	NM_133232	fwd	CTGACTCGCTACCTCAACTG
		rev	ACTGTTTTCGGACTCTCATGG
Mpgk1	NM_008828	fwd	AACCTCCGCTTTCATGTAGAG
		rev	GACATCTCCTAGTTTGGACAGTG
Mcd36/scarb3	NM_007643	fwd	GCGACATGATTAATGGCACAG
		rev	GATCCGAACACAGCGTAGATAG
Mhk1	NM_010438	fwd	TCACATTGTCTCCTGCATCTC
		rev	CTTTGAATCCCTTTGTCCACG
Hvcn1-1	NM_28240	fwd	ATGACTTCCCATGACCCAAAGG
		rev	TCGTCCCCAACCACCGTAA
**Mitochondrial DNA *MT-16s***	NM_34377	fwd	CCGCAAGGGAAAGATGAAAGAC
		rev	TCGTTTGGTTTCGGGGTTTC
**Mitochondrial DNA *Nd1***	NM_7455	fwd	CTAGCAGAAACAAACCGGGC
		rev	CCGGCTGCGTATTCTACGTT
**Nuclear DNA *Hk2***	NM_4923	fwd	GCCAGCCTCTCCTGATTTTAGTGT
		rev	GGGAACACAAAAGACCTCTTCTGG
